# CAST/ELKS Proteins Control Voltage-Gated Ca^2+^ Channel Density and Synaptic Release Probability at a Mammalian Central Synapse

**DOI:** 10.1016/j.celrep.2018.06.024

**Published:** 2018-07-10

**Authors:** Wei Dong, Tamara Radulovic, R. Oliver Goral, Connon Thomas, Monica Suarez Montesinos, Debbie Guerrero-Given, Akari Hagiwara, Travis Putzke, Yamato Hida, Manabu Abe, Kenji Sakimura, Naomi Kamasawa, Toshihisa Ohtsuka, Samuel M. Young

**Affiliations:** 1 Department of Anatomy and Cell Biology, University of Iowa, Iowa City, IA 52242, USA; 2 Department of Otolaryngology, University of Iowa, Iowa City, IA 52242, USA; 3 Iowa Neuroscience Institute, University of Iowa, Iowa City, IA 52242, USA; 4 Aging Mind Brain Initiative, University of Iowa, Iowa City, IA 52242, USA; 5 Research Group Molecular Mechanisms of Synaptic Function, Max Planck Florida Institute for Neuroscience, Jupiter, FL 33458, USA; 6 Max Planck Florida Institute for Neuroscience Electron Microscopy Facility, Max Planck Florida Institute for Neuroscience, Jupiter, FL 33458, USA; 7 Key Laboratory of Medical Electrophysiology, Ministry of Education, Collaborative Innovation Center for Prevention and Treatment of Cardiovascular Disease, Institute of Cardiovascular Research, Southwest Medical University, Luzhou 646000, China; 8 Department of Biochemistry, University of Yamanashi, Yamanashi 409-3898, Japan; 9 Department of Cellular Neurobiology, Brain Research Institute, Niigata University, Niigata 951-8585, Japan; 11 Lead Contact

## Abstract

In the presynaptic terminal, the magnitude and location of Ca^2+^ entry through voltage-gated Ca^2+^ channels (VGCCs) regulate the efficacy of neurotransmitter release. However, how presynaptic active zone proteins control mammalian VGCC levels and organization is unclear. To address this, we deleted the CAST/ELKS protein family at the calyx of Held, a Ca_V_2.1 channel-exclusive presynaptic terminal. We found that loss of CAST/ELKS reduces the Ca_V_2.1 current density with concomitant reductions in Ca_V_2.1 channel numbers and clusters. Surprisingly, deletion of CAST/ELKS increases release probability while decreasing the readily releasable pool, with no change in active zone ultrastructure. In addition, Ca^2+^ channel coupling is unchanged, but spontaneous release rates are elevated. Thus, our data identify distinct roles for CAST/ELKS as positive regulators of Ca_V_2.1 channel density and suggest that they regulate release probability through a post-priming step that controls synaptic vesicle fusogenicity.

## In Brief

Dong et al. show that CAST/ELKS have multiple roles in presynaptic function. These proteins positively regulate Ca_V_2.1 channel abundance and negatively regulate release probability. The authors propose that CAST/ELKS regulate release probability at a step in synaptic vesicle release that regulates the energy barrier for synaptic vesicle fusion.

## Graphical Abstract

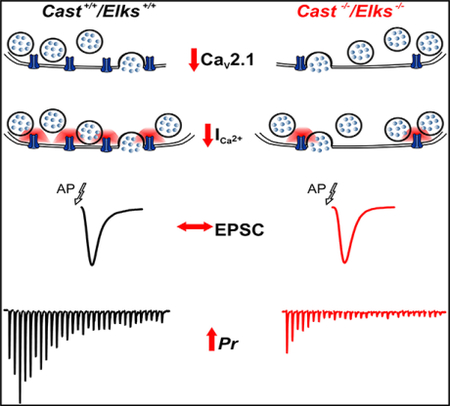

## INTRODUCTION

The density of voltage-gated Ca^2+^ channels (VGCCs) and their proximity relative to synaptic vesicles (SVs), defined as coupling, in the presynaptic terminal are critical determinants of SV release probability (*P*_*r*_) and kinetics (Eggermann et al., 2011). Within the presynaptic active zone (AZ), a dense network of proteins, the cytomatrix of the active zone (CAZ) is implicated in regulating VGCC function. In mammals, a core AZ component is the large multidomain CAST/ELKS protein family, which is encoded by two genes, *Erc1* (CAST2/ELKS1/Rab6IP2A protein [ELKS]) and *Erc2* (CAST/ELKS2 protein [CAST]) (Deguchi-Tawarada et al., 2004; Ohtsuka et al., 2002; Wang et al., 2002), that are the orthologs of *D. melanogaster Bruchpilot* (Wagh et al., 2006) and *C. elegans Elks* (Deken et al., 2005). CAST/ELKS are highly conserved; interact with multiple AZ proteins, including RIMs, Munc13s, Bassoon/Piccolo, liprin-α, and the Ca_V_2.1 α_1_ subunit; and control Ca_V_2.1 channel (Ca_V_2.1) voltage-dependent activation via a Ca_V_β4 subunit interaction (Kiyonaka et al., 2012). In mice, ELKS deletion is embryonically lethal (Liu et al., 2014). CAST deletion leads to disparate phenotypes with reduced photoreceptor synapse size (tom Dieck et al., 2012). There are either increased effects on *P*_*r*_ (Kaeser et al., 2009) or no effects on *P*_*r*_ (Kobayashi et al., 2016) in hippocampal γ-aminobutyric acid (GABAergic) neurons, while there are reductions in *P*_*r*_ (Kobayashi et al., 2016) or no effect on *P*_*r*_ (Kaeser et al., 2009) in hippocampal glutamatergic neurons. Combined deletion of CAST/ELKS reduced Ca^2+^ influx and *P*_*r*_ in GABAergic neurons (Liu et al., 2014), but it did so with no change in Ca^2+^ influx or *P*_*r*_ in glutamatergic neurons (Held et al., 2016).

Our understanding of CAST/ELKS function is largely based on heterogeneous populations of glutamatergic or GABAergic synapses from hippocampal neurons (Held et al., 2016; Kaeser et al., 2009; Kobayashi et al., 2016; Liu et al., 2014), which are in different developmental states, use different release modalities, and contain multiple Ca_V_2 subtypes (Eggermann et al., 2011). Therefore, the regulatory roles of CAST/ELKS in Ca^2+^ entry and their impact on SV release in mammalian presynaptic terminals remain unclear. To address these questions, we used the calyx of Held, a fast-firing Ca_V_2.1-exclusive synapse to precisely define presynaptic mechanisms of Ca_V_2.1 regulation and SV release (Borst and Soria van Hoeve, 2012). We used *Cast/Erc2* knockout (*Cast* KO), *Elk1s/Erc1* conditional (*Elks* CKO) (*Cast*^−/−^/*Elks*^*fl/fl*^) transgenic mice, in conjunction with conditional ablation of ELKS in the calyx of Held, to analyze CAST/ELKS presynaptic regulatory roles. We show that deletion of CAST/ELKS significantly decreased Ca_V_2.1 currents and numbers, shifted voltage dependence of activation to more positive potentials, and reduced the readily releasable pool (RRP) size but did so with no change in AZ ultrastructure. Counterintuitively, we found that loss of CAST/ELKS increased *P*_*r*_. Paired recordings revealed no change in SV release kinetics, while spontaneous release rates were increased and resting intraterminal [Ca^2+^] was unchanged. Overall, our results suggest that CAST/ELKS regulate *P*_*r*_ through post-priming, a late step controlling SV release. Based on our results, we conclude that CAST/ELKS have multiple regulatory roles essential to the regulation of synaptic transmission.

## RESULTS

### Loss of CAST/ELKS Affects Ca_V_2.1 Current Density and Activation

**Figure 1.**
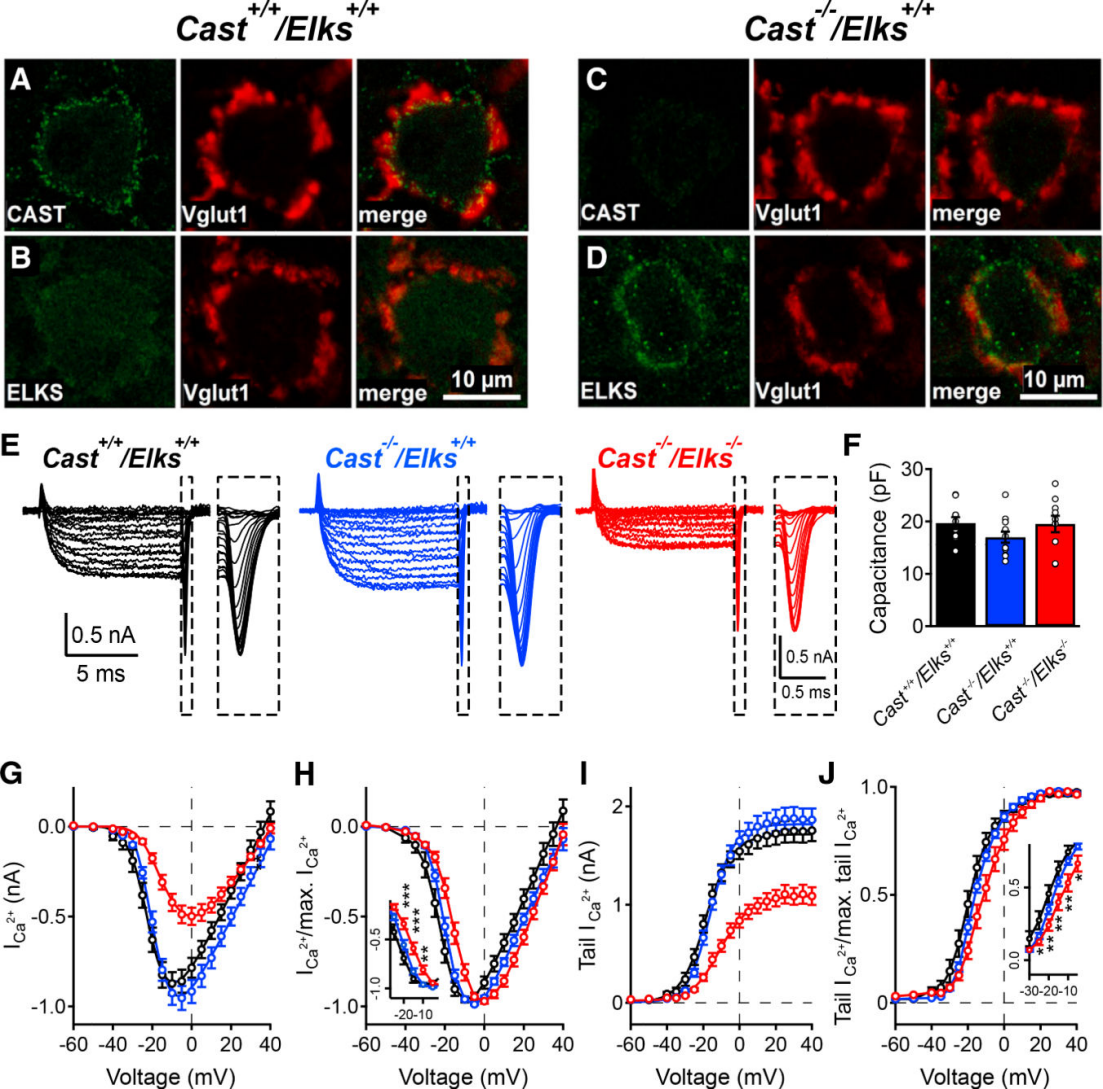
Increased ELKS Levels in the Absence of CAST, while Loss of CAST/ELKS Leads to a Reduction of Ca_V_2.1 Currents (A–D) IHC staining for CAST (green) and VGlut1 (red) in the P18 *Cast*^+/+^/*Elks*^+/+^ (A) and *Cast*^−/−^/*Elks*^+/+^ (C) calyces of the Held/MNTB synapse. IHC staining for ELKS (green) and VGlut1 (red) in the P18 *Cast*^+/+^/*Elks*^+/+^ (B) and *Cast*^−/−^/*Elks*^+/+^ (D) calyces of the Held/MNTB synapse. (E) Representative traces of Ca^2+^ currents recorded from *Cast*^+/+^/*Elks*^+/+^, *Cast*^−/−^/*Elks*^+/+^, and *Cast*^−/−^/*Elks*^−/−^ calyces from P16 to P19. Inset: enlarged tail currents. (F) Mean cell capacitance. (G) Mean peak Ca^2+^ currents as functions of voltage. (H) Mean peak Ca^2+^ currents normalized to the maximum peak as functions of voltage. Inset: voltage range −25 to −5 mV. (I) Peak Ca^2+^ tail currents as functions of voltage. (J) Peak Ca^2+^ tail currents normalized to the maximum tail current as functions of voltage. Inset: voltage range −30 to −5 mV. Presynaptic recordings: *Cast*^+/+^/*Elks*^+/+^, n = 10; *Cast*^−/−^/*Elks*^+/+^, n = 12; *Cast*^−/−^/*Elks*^−/−^, n = 10; **p < 0.01, ***p < 0.001; one-way ANOVA. Data are represented as mean ± SEM.

Because the CAST and ELKS expression pattern at the calyx was unknown, we performed immunohistochemistry (IHC) in conjunction with confocal microscopy on *Cast*^+/+^/*Elks*^+/+^ and *Cast*^−/−^/*Elks*^+/+^ calyces (Figure 1). In *Cast*^+/+^/*Elks*^+/+^ calyces, CAST was detected while ELKS was undetectable (Figures 1A and 1B). However, analysis on the *Cast*^−/−^/*Elks*^+/+^ calyces (Figures 1C and 1D) revealed prominent ELKS expression and demonstrate that ELKS levels are elevated in the absence of CAST at the calyx.

CAST/ELKS are implicated in regulating Ca_V_2.1 voltage-dependent activation in a recombinant cell line (Kiyonaka et al., 2012), while loss of CAST/ELKS reduced Ca_V_2-mediated Ca^2+^ influx in primary cultured inhibitory neurons (Liu et al., 2014). However, the mechanisms that underpin reductions in Ca^2+^ influx remain unclear; they could be due to changes in Ca_V_2 voltage-dependent activation or reductions in Ca_V_2 numbers at the presynaptic terminal. To ascertain CAST/ELKS regulation of Ca_V_2.1 function, we injected helper-dependent adenovirus (HdAd) vectors that independently co-expressed Cre and EGFP in the cochlear nucleus (Lübbert et al., 2017) of the *Cast* KO, *Elks* CKO mouse line (Figures S1 and S2) to ablate both CAST and ELKS at the calyx. Subsequently, we measured the Ca^2+^ current-voltage (IV) relationship in *Cast*^+/+^/*Elks*^+/+^, *Cast*^−/−^/*Elks*^+/+^, and *Cast*^−/−^/*Elks*^−/−^ calyces using direct presynaptic whole-cell patch-clamp recordings (Figure 1E). Analysis of the IV data revealed that the loss of both CAST and ELKS resulted in a significant decrease of peak I_Ca_, a rightward shift in the slope of the voltage dependence of activation, and no change in *C*_*slow*_ values (Figures 1F–1H). Analysis of Ca^2+^ tail current peak amplitudes confirmed a reduction in I_Ca_, as well as a rightward shift in activation (Figures 1I and 1J). Based on these data, we demonstrate that CAST/ELKS regulate Ca_V_2.1 current levels and voltage dependence of activation.

### CAST/ELKS Regulate Ca_V_2.1 Numbers and Organization at the Putative AZ

**Figure 2.**
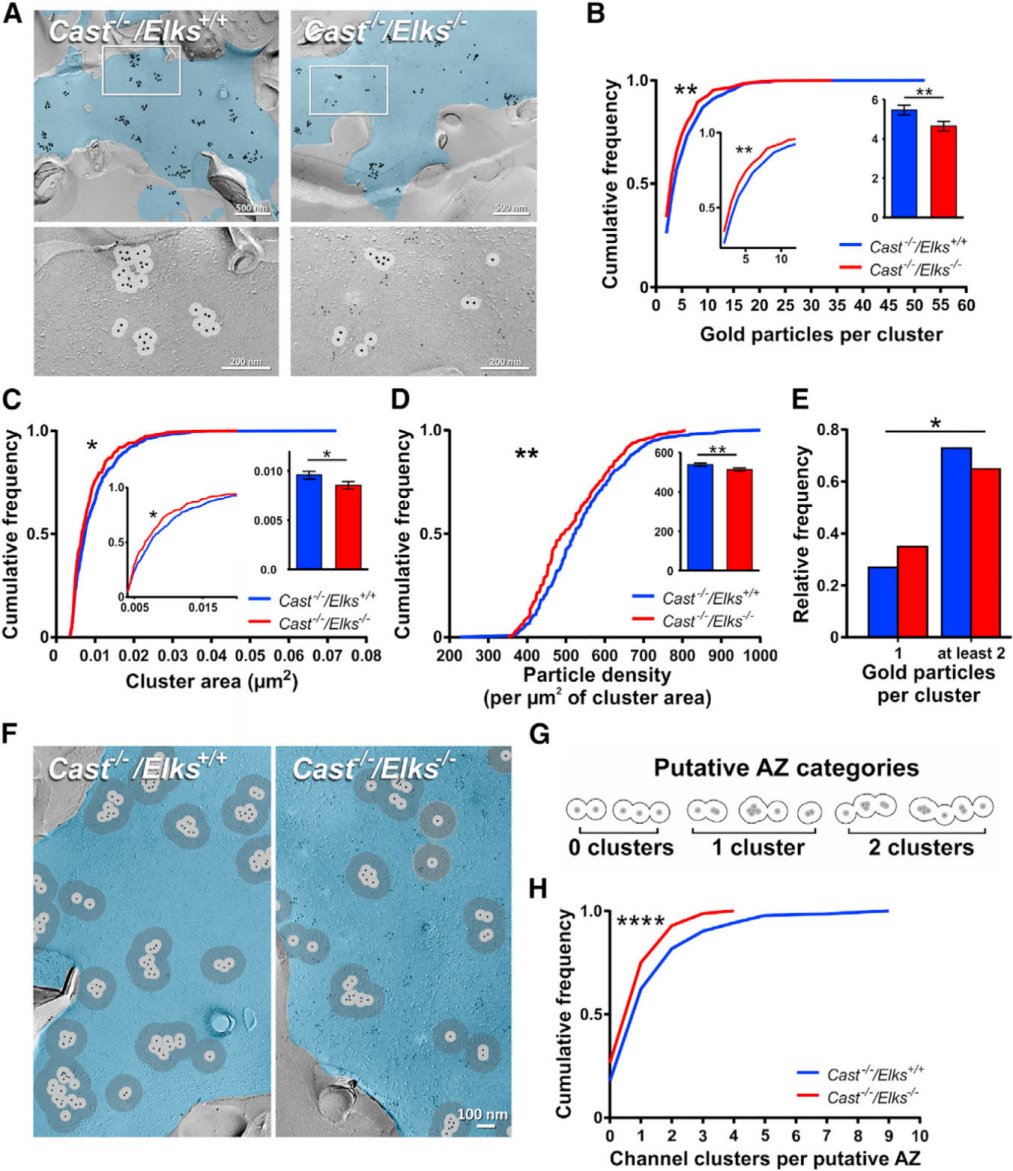
Loss of CAST/ELKS Leads to a Decrease in Ca_V_2.1 Channels and Cluster at the Putative AZ (A) Representative SDS-FFRL replicas of calyx P faces (pseudocolored blue) of *Cast*^−/−^/*Elks*^+/+^ and *Cast*^−/−^/*Elks*^−/−^ mice at P18. Top: Ca_V_2.1 distribution. Large gold particles (12 nm) label Ca_V_2.1 (enlarged for visibility); small gold particles (6 nm) label myristolated enhanced green fluorescent protein (mEGFP) (*Cast*^−/−^/*Elks*^−/−^ only). Bottom: high-magnification images of the boxed areas showing clustering of gold particles. EM images are montages of multiple tiles assembled from the calyx P face area containing Ca_V_2.1 clusters (B) Cumulative frequency distribution of gold particles per cluster. (C) Cumulative frequency distribution of the cluster area. (D) Cumulative frequency distribution of the gold particle density per square micrometer of cluster area. Inset: bar graph of data. (E) Comparison of the relative frequency of single channels to channel clusters at P18. (F) Representative SDS-FFRL replicas of calyx P faces (blue) of *Cast*^−/−^/*Elks*^+/+^ and *Cast*^−/−^/*Elks*^−/−^ mice at P18 measuring putative AZ. (G) Diagram of putative AZ categories (gray circles). (H) Cumulative frequency distribution of the channel cluster assembly within a putative AZ. *p < 0.05, **p < 0.01; Mann-Whitney U test and Fisher’s exact test. Data are represented as cumulative frequency, relative frequency, and mean ± SEM.

To determine whether the Ca_V_2.1 current reductions were due to a loss of Ca_V_2.1 channel numbers, we carried out SDS-digested freeze-fracture replica immunogold labeling (SDS-FFRL) on the *Cast*^−/−^/*Elks*^+/+^ and *Cast*^−/−^/*Elks*^−/−^ calyces (Figure 2). *Cast*^−/−^/*Elks*^+/+^ was used as control because the calcium current (I_Ca_) was unchanged compared to wild-type and served as an in-slice control to minimize potential sample-to-sample variability. Using 30 nm radius circles (Althof et al., 2015), we found that loss of CAST/ELKS resulted in a significant reduction in the mean number of gold particles per cluster compared to control (4.7 ± 0.21 versus 5.5 ± 0.22, p < 0.05), cluster area (0.0086 ± 0.0003 versus 0.0097 ± 0.0003 μm^2^, p < 0.05), and particle density in a cluster (Figures 2A–2D; Table S2) and a selective reduction in clusters with ≥11 particles. We also found a change in relative distribution of single gold particles not found in a cluster (27% versus 35%, p = 0.0053) (Figure 2E).

Because AZs can contain multiple VGCC clusters (Budisantoso et al., 2012; Holderith et al., 2012), it was important to determine whether cluster numbers within individual AZs changed in the absence of CAST/ELKS. At the calyx, use of a 100 nm radius to analyze Ca_V_2.1 clustering corresponds to an average AZ area in serial section electron microscopy (EM) (Sätzler et al., 2002; Taschenberger et al., 2002) and a group of channels with the optimal cluster rate (Nakamura et al., 2015). Using a 100 nm radius, we counted the Ca_V_2.1 clusters within the putative AZ area (Figures 2F and 2G). Although single particles could be found within the AZ area, they were not counted as a cluster (Figure 2G). We found a reduction in putative AZ area (0.07 ± 0.002 versus 0.096 ± 0.004 μm^2^, p < 0.001) and mean number of Ca_V_2.1 clusters within the putative AZ in *Cast*^−/−^/*Elks*^−/−^ calyces compared to *Cast*^−/−^/*Elks*^+/+^ calyces (1.07 ± 0.05 versus 1.6 ± 0.09, p < 0.0001) (Figure 2H; Table S2) and a specific loss in AZs that contain 5 or more clusters in the *Cast*^−/−^/*Elks*^−/−^ calyces (Figure 2H). Altogether, our results revealed that Ca_V_2.1 current reduction was correlated with a decrease in the Ca_V_2.1 numbers per cluster and cluster number per putative AZ. Therefore, we conclude that CAST/ELKS regulate Ca_V_2.1 numbers and organization at the AZ.

### Loss of CAST/ELKS Does Not Affect Basal AP-Evoked Synaptic Transmission

**Figure 3.**
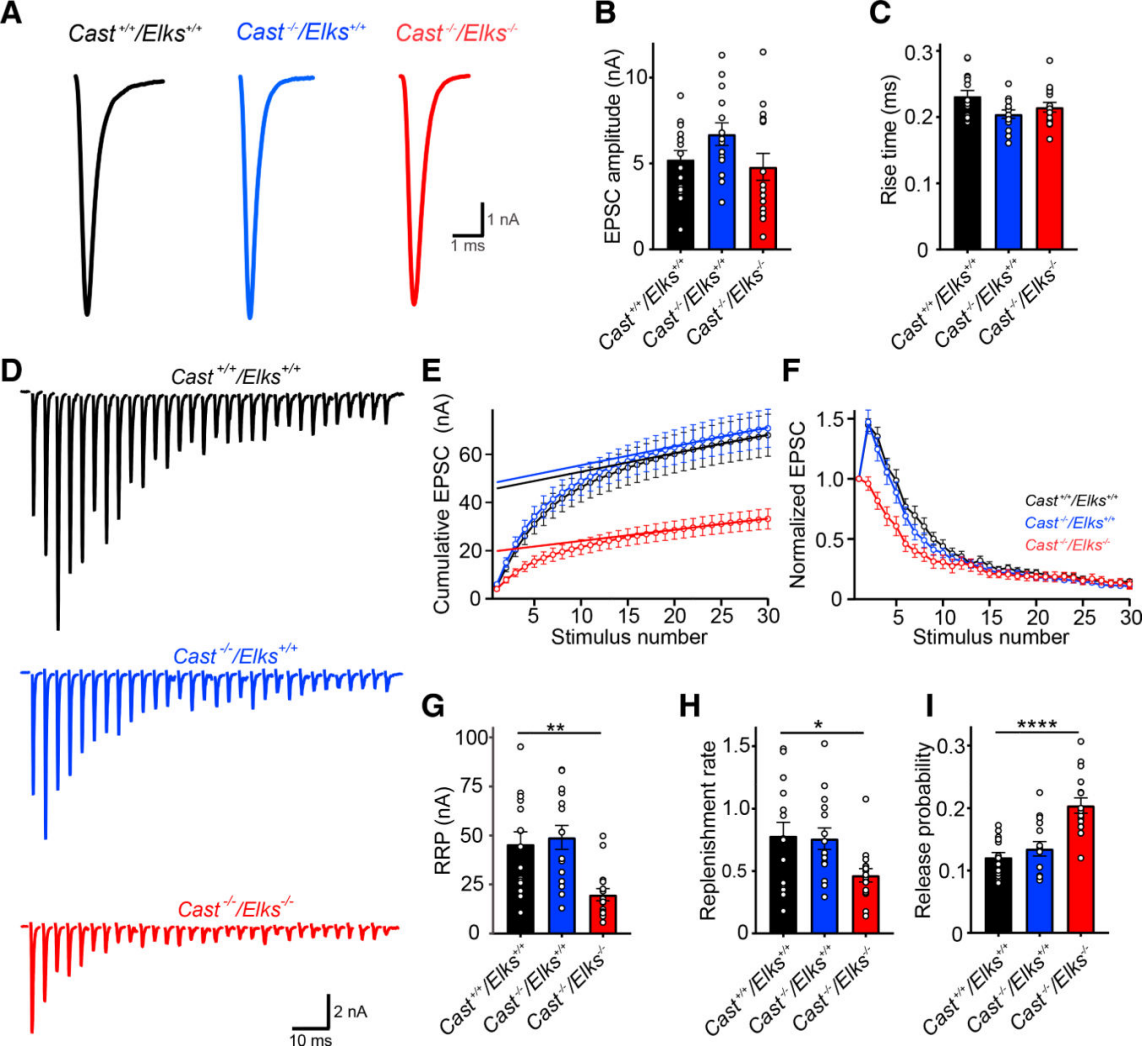
Loss of CAST/ELKS Results in Smaller RRP, Slower Replenishment Rate, and Increased *P_r_* but No Changes in Basal Synaptic Transmission (A) Representative traces of single AP-evoked EPSC of *Cast*^+/+^/*Elks*^+/+^ (black), *Cast*^−/−^/*Elks*^+/+^ (blue), and *Cast*^−/−^/*Elks*^−/−^ (red). (B) Basal EPSC amplitudes. (C) 10–90 risetimes. (D) Representative EPSCs at 300 Hz (30 stimuli). (E) Cumulative EPSC amplitudes. (F) Normalized EPSC amplitude to the first EPSC. (G) Mean RRP sizes. (H) Replenishment rate. (I) Release probability. *Cast*^+/+^/*Elks*^+/+^, n = 15; *Cast*^−/−^/*Elks*^+/+^, n = 15; *Cast*^−/−^/*Elks*^−/−^, n = 16; *p < 0.05, **p < 0.01, ***p < 0.001, ****p < 0.0001; one-way ANOVA. Data are represented as mean ± SEM.

To determine how the reductions in Ca_V_2.1 numbers and clusters affected action potential (AP)-evoked release, we performed afferent fiber stimulation of calyx axons and recorded α-amino-3-hydroxy-5-methyl-4-isoxazolepropionic acid (AMPA) receptors (AMPAR) excitatory postsynaptic currents (EPSCs) from the medial nucleus of the trapezoid body (MNTB) principal cells, innervated by transduced or non-transduced calyces using various genetic backgrounds. To uncover how basal AP-evoked release was affected, we used a 0.05 Hz stimulation in 1.2 mM [Ca^2+^] external to mimic *in vivo P*_*r*_ (Lorteije et al., 2009). Surprisingly, we found that loss of both CAST and ELKS had no effect on basal synaptic transmission compared to wild-type (Figures 3A–3C), indicating that reductions in Ca_V_2.1 numbers and clusters in the AZ do not affect basal synaptic transmission.

### CAST/ELKS Regulate Initial *P*_*r*_, RRP Size, and Replenishment

CAST/ELKS are negative regulators (Kaeser et al., 2009) or positive regulators of RRP size and *P*_*r*_ (Held et al., 2016; Liu et al., 2014). Thus, it was possible that despite Ca_V_2.1current reduction, the lack of effect on basal AP-evoked synaptic transmission was due to an increased RRP size or *P*_*r*_. To determine whether there was an increased RRP size or *P*_*r*_ in the *Cast*^−/−^/*Elks*^−/−^ calyces, we carried out afferent fiber stimulation at 300 Hz. First, we determined the RRP size by plotting the cumulative EPSC amplitude versus stimulus number and then back-extrapolating with a line fit (Figure 3E) (Neher, 2015). Analysis of EPSCs from *Cast*^−/−^/*Elks*^−/−^ compared to the *Cast*^+/+^/*Elks*^+/+^ calyces in response to 300 Hz stimulation revealed a 2-fold reduction in the RRP size (19.82 ± 3.12 versus 45.59 ± 6.3, p = 0.0022) and a slower replenishment rate (0.47 ± 0.05 versus 0.78 ± 0.11, p = 0.044) (Figures 3G and 3H; Table S3). Calculation of initial *P*_*r*_ (first EPSC divided by the calculated RRP size) (Figure 3I), revealed a ~2-fold increase in initial *P*_*r*_ in *Cast*^−/−^/*Elks*^−/−^ calyces (0.2 ± 0.012 versus 0.12 ± 0.008, p = 0.0001) but no difference between *Cast*^−/−^/*Elks*^+/+^ and *Cast*^+/+^/*Elks*^+/+^ calyces. Normalization revealed a dramatic alteration in frequency-dependent plasticity with an absence of facilitation and an increased rate of depression only in the *Cast*^−/−^/*Elks*^−/−^ calyces (Figure 3F; Table S3). To test whether ELKS is the critical isoform at the calyx, we carried out afferent fiber stimulation with *Cast*^+/+^/*Elks*^−/−^ calyces and found no differences in synaptic transmission compared to *Cast*^+/+^/*Elks*^+/+^ calyces (Figure S3). These results show that deletion of CAST/ELKS increases initial *P*_*r*_ but with a reduction in the RRP size and replenishment rates. We conclude that increased *P*_*r*_ is the cause for no change in basal AP-evoked synaptic transmission.

### Loss of CAST/ELKS Does Not Change the RRP or Total Release Pool Release Kinetics but Reduces the Total Releasable Pool Size

**Figure 4.**
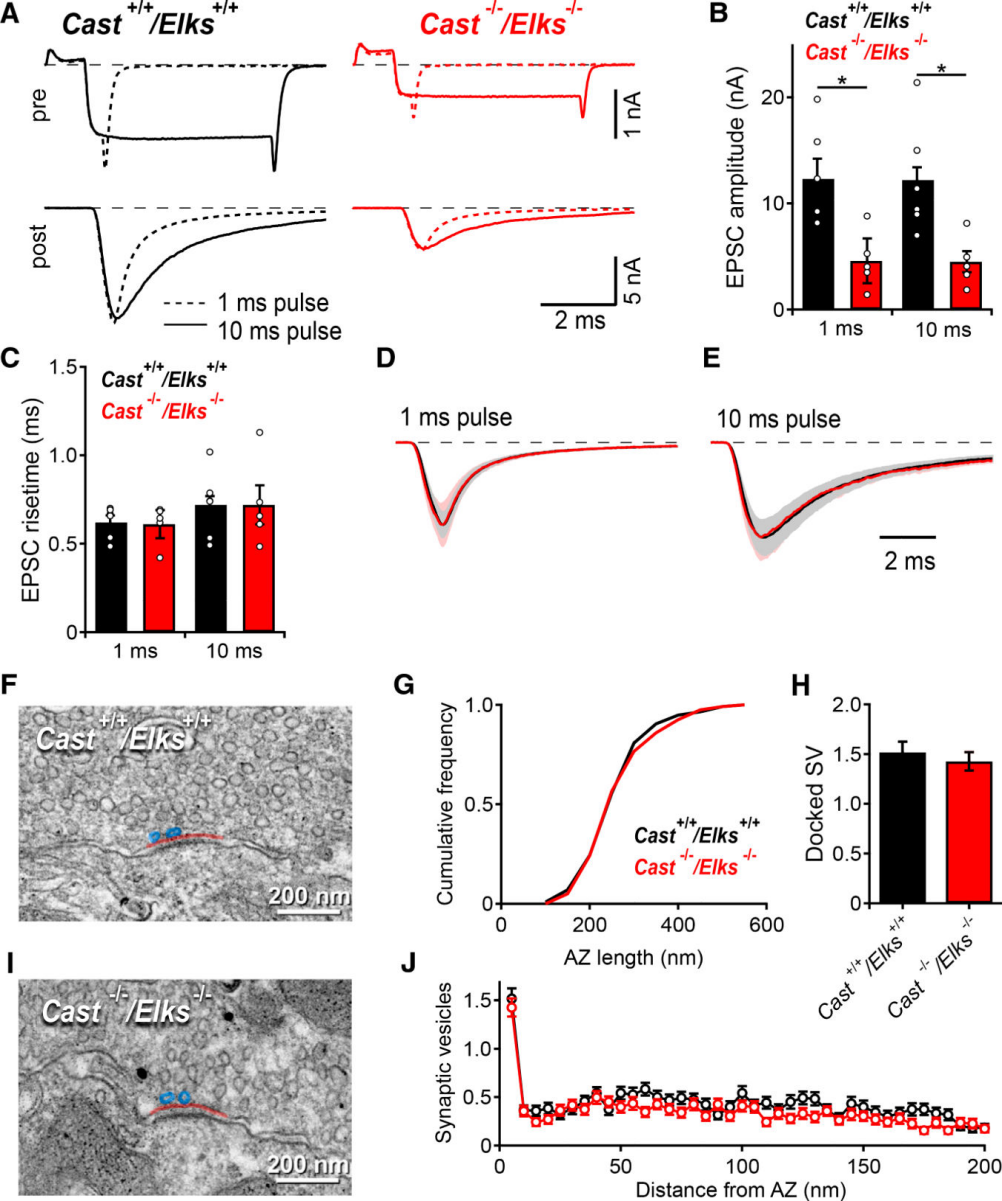
Loss of CAST/ELKS Causes Decreased EPSC Amplitude and Increased mEPSC Frequency but No Changes in EPSC Risetime, AZ Length, or Vesicle Docking (A) Top: average presynaptic Ca^2+^ current evoked by 1 ms (dotted line) or 10 ms (continuous line) depolarizations. Bottom: averaged postsynaptic AMPAR currents recorded simultaneously. (B) Mean EPSC amplitude. (C) Mean EPSC risetime. (D) Mean EPSC normalized to peak current for 1 ms. (E) 10 ms presynaptic depolarizations. (F–I) Representative electron micrographs of AZs taken from *Cast*^+/+^/*Elks*^+/+^ (F) and *Cast*^−/−^/*Elks*^−/−^ (I) calyces at P18. (G) AZ length plotted as the cumulative frequency distribution. (H) Number of docked SVs per AZ. (J) Distribution of synaptic vesicles within a 0 to 200 nm distance from the AZ in 5 nm bins. Paired recordings: *Cast*^+/+^/*Elks*^+/+^, n = 6; *Cast*^−/−^/*Elks*^+/+^, n = 5; *p < 0.05, ***p < 0.001; t test. Data are represented as mean ± SEM.

Because CAST/ELKS deletion leads to an increased initial *P*_*r*_ despite reduced Ca_V_2.1 currents, we hypothesized the increased *P*_*r*_ was due to either tighter SV coupling or changes in post-priming, which affects SV fusogenicity (Neher, 2017). The paired whole-cell patch-clamp configuration at the calyx of Held synapse can be used to measure changes in SV coupling distances (Neher and Sakaba, 2008) and results in a loss of signaling cascades that control post-priming (Lou et al., 2005). Therefore, we performed paired whole-cell voltage-clamp recordings to distinguish the mechanisms controlling the *P*_*r*_ increase (Figure 4). We applied a 1 ms step depolarization and then a 10 ms step depolarization at the post-natal day (P) 16–P19 calyx with 5 mM EGTA in the presynaptic patch pipettes (Chen et al., 2015). The 1 ms step depletes SVs within 25 nm of Ca_V_2.1, the RRP for AP-evoked release (fast pool), while the 10 ms step pulse depletes the entire pool of fusion-competent SVs within 100 nm of Ca_V_2.1, the total releasable pool (Chen et al., 2015).

In response to the 1 and 10 ms depolarizations, we found a significant decrease in Ca^2+^ charge and peak EPSC amplitudes (Figures 4A and 4B; Table S4) between the *Cast*^+/+^/*Elks*^+/+^ and the *Cast*^−/−^/*Elks*^−/−^ genotypes. Because peak EPSC 10–90 risetimes correlate with changes in coupling distances (Neher and Sakaba, 2008), we measured the 1 and 10 ms EPSC 10–90 risetimes and found no difference (Figure 4C). Overlay of the EPSC waveforms normalized to the EPSC peak revealed no change in release kinetics in the *Cast*^−/−^/*Elks*^−/−^ calyces (Figures 4D and 4E). Analysis of the 1:10 ms ratio indicated no change in the ratio of RRP size to total releasable pool size (Table S4). Therefore, we conclude that the increased *P*_*r*_ in the absence of CAST/ELKS is due not to changes in Ca_V_2.1 to SV coupling but rather to changes in post-priming.

Because the number of docked SVs correlates with the RRP (Schikorski and Stevens, 2001), we analyzed transmission electron microscopy (TEM) images from *Cast*^+/+^/*Elks*^+/+^ and *Cast*^−/−^/*Elks*^−/−^ calyces to assess whether the RRP size and total releasable pool size reductions were due to a reduced number of docked SVs. Close analysis of SV distribution revealed no change in docked SV numbers, SVs within 5 nm (Taschenberger et al., 2002), SV distribution, or AZ length in *Cast*^−/−^/*Elks*^−/−^ versus *Cast*^+/+^/*Elks*^+/+^ (Figures 4F–4J; Table S2). Altogether, we demonstrate that the reductions in the RRP size and total releasable pool size are not due to reduced SV docking.

### Loss of CAST/ELKS Leads to an Increase in Spontaneous Release Rate

**Figure 5.**
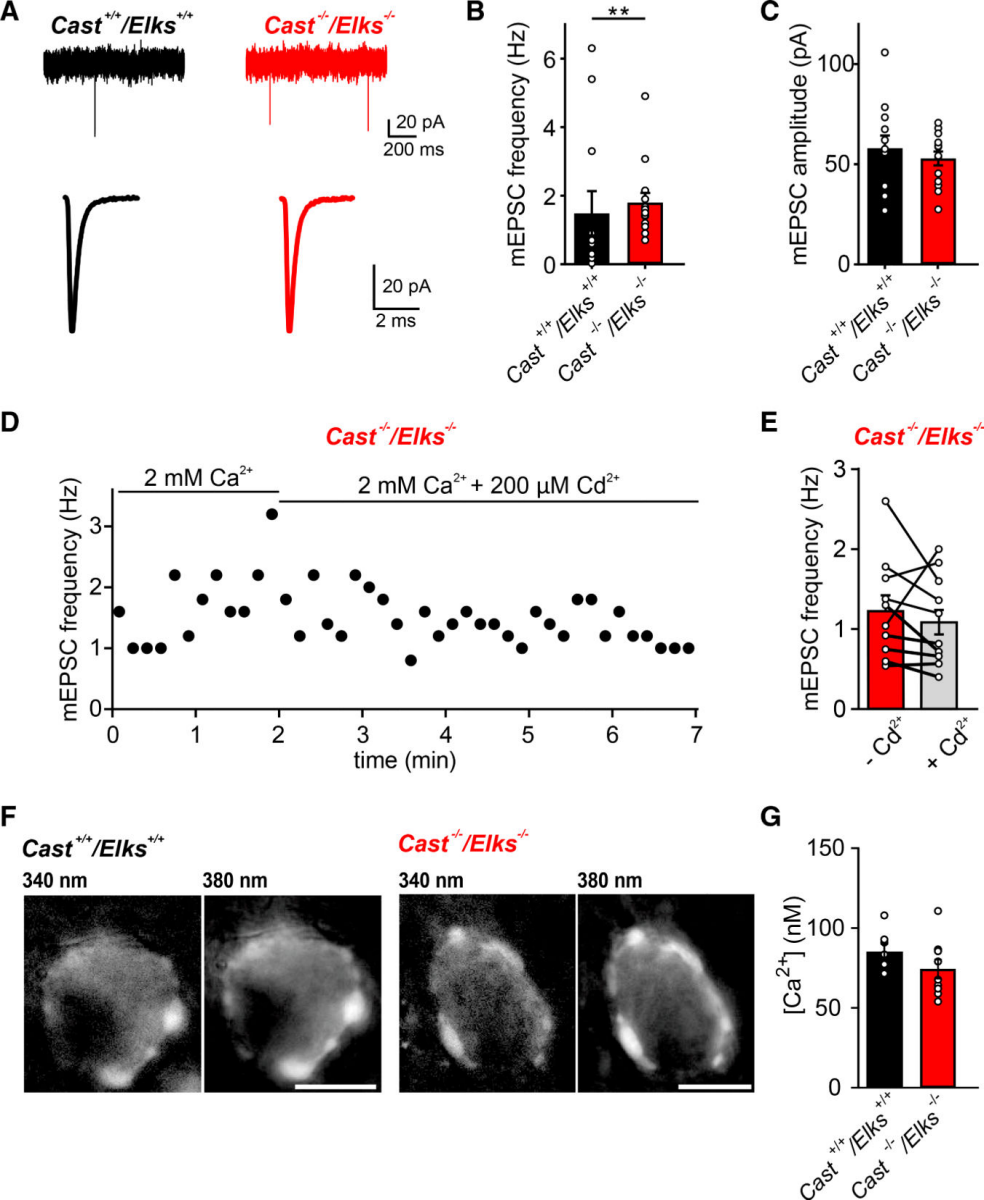
Loss of CAST/ELKS Results in Increased Spontaneous Release Rates (A) Top: representative recordings of mEPSCs from *Cast*^+/+^/*Elks*^+/+^ (black) and *Cast*^−/−^/*Elks*^−/−^ (red). Bottom: average mEPSC waveform from the same cell. (B) mEPSCs population data show increased frequency in *Cast*^−/−^/*Elks*^−/−^. (C) Mean amplitude of mEPSCs from individual cells. (D) Representative example of mEPSC frequency during Cd^2+^ application. (E) Mean frequency of mEPSCs before and after Cd^2+^ application (−Cd^2+^ and +Cd^2+^, respectively). (F) Example images of *Cast*^+/+^/*Elks*^+/+^ and *Cast*^−/−^/*Elks*^−/−^ presynaptic terminals filled with 100 mM Fura-2 and excited at 340 nm (left) or 380 nm (right). Scale bar, 10 μm. (G) Basal [Ca^2+^] for *Cast*^+/+^/*Elks*^+/+^ and *Cast*^−/−^/*Elks*^−/−^ terminals, respectively. In (A)–(C), *Cast*^+/+^/*Elks*^+/+^, n = 12; *Cast*^−/−^/*Elks*^−/−^, n = 14; **p < 0.01; t test. In (E), n = 10; paired t test. In (G), *Cast*^+/+^/*Elks*^+/+^, n = 7; *Cast*^−/−^/*Elks*^−/−^, n = 9. Data are represented as mean ± SEM.

Because the calyx containsmultipleAZs in parallel (Borst and Soria van Hoeve, 2012), the RRP and total releasable pool size reductions could be due to a reduction in the total number of AZs or the number of AZs that support Ca^2+^-evoked release. In both cases, similar reductions in Ca^2+^ influx would be observed in our presynaptic recordings. Spontaneous release can be regulated independently from evoked release mechanisms (Kavalali, 2015) and does not require VGCCs (Williams and Smith, 2018). Furthermore, their frequency is correlated with the number of AZs that contain fusion-competent SVs (Kavalali, 2015) and changes in post-priming that affect SV fusogenicity, with increased SV fusogenicity correlating with higher spontaneous release rates (Basu et al., 2007). Therefore, to determine whether increased *P*_*r*_ was due to changes in post-priming and whether there was a decrease in the number of AZs or the AZs that supported evoked release, we analyzed miniature excitatory postsynaptic currents (mEPSCs) from *Cast*^+/+^/*Elks*^+/+^ and *Cast*^−/−^/*Elks*^−/−^ synapses (Figure 5). We found an increase in the mEPSC frequency in the *Cast*^−/−^/*Elks*^−/−^ synapses versus the *Cast*^+/+^/*Elks*^+/+^ calyces (Figure 5B; Table S5) (1.55 versus 0.45, median) and no change in quantal amplitude (Figure 5C; Table S5). In addition, we found that the mEPSCs were VGCC independent, because blocking with 200 μM Cd^2+^ had no effect on mEPSC frequency in *Cast*^−/−^/*Elks*^−/−^ synapses (Figures 5D and 5E). Finally, we measured the resting intraterminal [Ca^2+^] in *Cast*^+/+^/*Elks*^+/+^ and *Cast*^−/−^/*Elks*^−/−^ calyces to determine whether this caused the increase in mEPSC frequency. Because there was no difference between the basal intraterminal [Ca^2+^] in *Cast*^+/+^/*Elks*^+/+^ calyces and that in *Cast*^−/−^/*Elks*^−/−^calyces(85.1±5versus 74.3 ± 6 nM) (Table S5), we can rule out that increased basal intraterminal [Ca^2+^] is not responsible for the increase in mEPSC frequency. Therefore, our data suggest that the loss of RRP and total releasable pool in the absence of CAST/ELKS is due to the loss of AZs that support Ca^2+^-evoked release, not a reduced AZ number, and that increased mEPSC frequency is due to changes in post-priming steps that increase SV fusogenicity.

### Loss of CAST/ELKS Proteins and Their Impact on Ca^2+^ Sensitivity of Basal AP-Evoked Transmitter Release

Post-priming affects SV fusogenicity by increasing SV release willingness (Basu et al., 2007; Wierda et al., 2007), which modulates the Ca^2+^ sensitivity of release independent of the Ca^2+^ sensor for synchronous release (Schotten et al., 2015). Because genetic manipulations can differentially affect post-priming (Schotten et al., 2015), we characterized how loss of CAST/ELKS affected the Ca^2+^ sensitivity of release (Figure S4). Our analysis revealed that there were no differences in EPSCs at all external [Ca^2+^], although there was a trend toward a smaller response in 2.0 mM in the *Cast*^−/−^/*Elks*^−/−^ calyces compared to *Cast*^+/+^/*Elks*^+/+^ (Figure S4). Therefore, our data suggest that loss of CAST/ELKS affects post-priming, which results in no apparent change in Ca^2+^ sensitivity of AP-evoked release at physiological Ca^2+^ levels.

## DISCUSSION

In this study, we uncovered roles for CAST/ELKS in regulating presynaptic function. We conclude that CAST/ELKS are positive regulators for controlling Ca_V_2.1 levels and organization in the presynaptic terminal. Furthermore, CAST/ELKS negatively regulate *P*_*r*_ through a post-priming step that affects SV fusogenicity.

Deletion of CAST resulted in altered phenotypes in inhibitory hippocampal synapses (Kaeser et al., 2009), in excitatory CA3-CA1 hippocampal synapses (Kobayashi et al., 2016), and at ribbon synapses (tom Dieck et al., 2012), but not at excitatory hippocampal synapses (Kaeser et al., 2009) and the calyx. Although CAST and ELKS are not mutually exclusive (Deguchi-Tawarada et al., 2004; Held et al., 2016; Kobayashi et al., 2016), data demonstrating ELKS is elevated in the *Cast*^−/−^ calyces are similar to previous observations that deletion of CAST leads to elevation of ELKS at hippocampal and photoreceptor synapses (Kobayashi et al., 2016; tom Dieck et al., 2012) and in *Cast*^−/−^ synaptic plasma membranes (Kobayashi et al., 2016). This suggests that ELKS levels are increased in specific synaptic populations to compensate for loss of CAST and that in certain synapses, ELKS is functionally equivalent to CAST. Thus, in synapses in which loss of CAST exhibits a phenotype, ELKS levels do not increase or the expressed ELKS isoform or isoforms cannot compensate for CAST.

### CAST/ELKS Regulation of Ca_V_2.1 Channels

Our results on CAST/ELKS regulation of Ca^2+^ entry at the calyx suggest that changes in Ca_V_2 voltage-dependent activation or reductions in Ca_V_2 levels underpin the reduction in Ca^2+^ entry in *Cast*^−/−^/*Elks*^−/−^ primary cultured inhibitory hippocampal synapses (Liu et al., 2014), even though this study reported no change in Ca_V_2 levels using confocal microscopy and western blot analysis using crude lysates. The discrepancy between our results and those of the prior study may be due to the difference of the techniques used, because the prior study did not measure Ca_V_2 levels at the presynaptic membrane and confocal microscopy lacks the resolution and quantitative ability of the EM techniques used in the present study. However, there is no evidence for a role for CAST/ELKS in regulation of Ca^2+^ entry at hippocampal glutamatergic synapses (Held et al., 2016). Differences between the two CAST/ELKS mouse models may also account for these discrepancies, because there are other phenotypic differences between the two CAST KO mice strains (Kaeser et al., 2009; Kobayashi et al., 2016). Because different isoforms of AZ proteins (Südhof, 2012), the Ca_V_2α_1_ subunits and Ca_V_β subunits (Lipscombe et al., 2013), are expressed in different synapses, it is likely that regulation of Ca_V_2 function by CAST/ELKS varies between synapses. However, our results are consistent with those found in *Drosophila*, in which deletion of the ortholog *Bruchpilot* leads to reductions in Ca^2+^ channel levels at the neuromuscular junction (NMJ) (Kittel et al., 2006), suggesting that CAST/ELKS regulation of Ca^2+^ channel levels is evolutionarily conserved.

The molecular mechanisms that control Ca_V_2.1 levels and organization within presynaptic terminals are unclear (Lübbert et al., 2017). *In vitro* biochemical assays have shown that CAST/ELKS directly interact with the domain II–III linker of the Ca_V_2.1 α_1_ subunit, with Ca_V_β4 (Kiyonaka et al., 2012), and with high affinity to the RIM1/2 PDZ domain (Lu et al., 2005). In addition, RIMs directly interact with Ca_V_β, while deletion of both RIM1 and RIM2 results in a reduction in presynaptic Ca_V_2 currents (Han et al., 2011), which is similar to the CAST/ELKS deletion phenotype. Because direct interactions between the Ca_V_2.1 α_1_ subunit and RIM1/2 are not essential for regulating Ca_V_2.1 channel levels within the presynaptic terminal (Lübbert et al., 2017), it is possible that RIM and CAST/ELKS interactions with Ca_V_b form a macromolecular complex that controls Ca_V_2.1 abundance though indirect or direct interactions. Finally, CAST/ELKS may regulate other Ca_V_2 isoform levels similar to RIMs at the prehearing calyx (Han et al., 2011).

### CAST/ELKS Regulation of *P*_*r*_

Despite a significant reduction in Ca_V_2.1 channels and a rightward shift in Ca_V_2.1 voltage-dependent activation, we found significant increase in *P*_*r*_. Independent of regulation of VGCCs, AZ proteins regulate *P*_*r*_ through distinct steps—Ca^2+^ sensing, coupling, and post-priming—thus regulating SV fusogenicity independent of Ca^2+^ sensor activity and coupling (Neher, 2017). Because we found increases in mEPSC frequency that were independent of VGCCs, even though our paired recording data revealed no change in RRP release kinetics and our calcium imaging measurements revealed no change in basal intraterminal [Ca^2+^], we hypothesize that CAST/ELKS regulate *P*_*r*_ through a post-priming step, not Ca^2+^ sensing or coupling. Post-priming has a time constant of ~4 s (Taschenberger et al., 2016); therefore, changes in post-priming in the *Cast*^−/−^/*Elks*^−/−^ calyces would be sufficient to explain no change in basal synaptic transmission, because this was measured at 0.05 Hz (20 s interstimulus time interval [ISI]). Post-priming steps are largely regulated by lipidic signaling that increases SV fusogenicity (Basu et al., 2007; Taschenberger et al., 2016; Wierda et al., 2007), although regulation of actin signaling cascades may also play a role (Lee et al., 2012; Montesinos et al., 2015). However, there is no evidence that CAST/ELKS directly regulate lipidic signaling cascades or the actin cytoskeleton. Therefore, CAST/ELKS may play either an indirect or a direct role in regulation of post-priming. Finally, CAST/ELKS regulation of post-priming appears not to be unique to the calyx, because CAST was previously implicated in regulating post-priming in inhibitory neurons (Kaeser et al., 2009).

Our findings indicate that the primary cause for the reduced RRP and total releasable pool sizes was significant loss of Ca_V_2.1 channels, not a loss of individual AZs. Our results are similar to previous findings in which CAST/ELKS null inhibitory hippocampal synapses had reduced Ca^2+^ entry and AP-evoked RRP but no change in the total releasable pool size measured in a Ca^2+^-independent manner(hypertonic sucrose) and the spontaneous release rate in low Ca^2+^ (Liu et al., 2014). Although the median spontaneous release rate in the *Cast*^−/−^/*Elks*^−/−^ calyces is elevated 3-fold compared to that in *Cast*^+/+^/*Elks*^+/+^ (1.5 versus 0.5 Hz), this is not underlying cause in the ~2-fold reduction in the RRP and total releasable pool, because the increased spontaneous release rate would only result in an RRP reduction size by one SV. Furthermore, the reduction in RRP size cannot be attributed to a change in mEPSC quantal amplitude, because we found no difference in mEPSC amplitudes in *Cast*^−/−^/*Elks*^−/−^ calyces compared to *Cast*^+/+^/*Elks*^+/+^ calyces. Our finding contrasts with a finding in *Cast*^−/−^ hippocampal synapses (Kobayashi et al., 2016), which reported an extremely minor but significant reduction in mEPSC amplitude (~1 pA) and can be attributed to differences between synapses, because these changes were not seen in inhibitory hippocampal synapses. Although we did not find a change in SV docking, we did not carry out high-pressure freezing, freeze substitution, and EM tomography, which has found SV docking defects that were not detected with chemical fixation (Imig et al., 2014; Siksou et al., 2007). Therefore, we cannot rule out a role for CAST/ELKS in SV docking. In addition, CAST/ELKS may regulate the RRP through other mechanisms independent of Ca_V_2.1 regulation, because loss of CAST/ELKS reduced RRP size independent of changes in Ca^2+^ entry in excitatory hippocampal neurons (Heldet al., 2016) or pancreatic β cells (Ohara-Imaizumi et al., 2005) or changes in bMunc13–2 levels (Kawabeetal.,2017),althoughbMunc13–2hasnotbeendetected at the calyx (Chen et al., 2013). Furthermore, because reductions in [Ca^2+^]_i_ affect SV replenishment (Hosoi et al., 2007; Wang and Kaczmarek, 1998), we cannot rule out a role for CAST/ELKS in the direct regulation of SV replenishment (Kobayashi et al., 2016; Mochida et al., 2016).

In summary, we show that CAST/ELKS are critical molecules for controlling Ca_V_2.1 function and organization and regulate *P*_*r*_. Future studies will resolve the molecular interactions and signaling pathways by which CAST/ELKS regulate VGCCs and synaptic transmission in different synapses and their roles in neuronal circuit function and impact on neurological diseases.

## STAR⋆METHODS

### CONTACT FOR REAGENT AND RESOURCE SHARING

Further information and requests for resources and reagents should be directed to and will be fulfilled by the Lead Contact Samuel M. Young, Jr. at samuel-m-young@uiowa.edu.

### EXPERIMENTAL MODEL AND SUBJECT DETAILS

#### Animals

All animals were used in accordance with all animal welfare laws approved by the Institutional Committee for Care and Use of Animals at the University of Yamanashi and the Max Planck Florida Institute for Neuroscience and University of Iowa. Both genders of animals were used for all experiments.

#### Generation of Erc1 (Elks) flox mice

Elks flox mice were generated as follows. We identified a bacterial artificial chromosome (BAC) clone, RP24–103F1 (BACPAC Resources Center) prepared from the C57BL/6 strain, carrying the entire coding sequence of *Erc1* (*Elks*) gene. The genomic DNA fragment carrying exon 11 was introduced into the pMC1DtpA (Taniguchi et al., 1997). The 1.8-kb DNA fragment carrying the 34-bp loxP sequence and phosphoglycerate kinase 1 (Pgk-1) promoter-driven neomycin phosphotransferase (neo) gene flanked by two Flp recognition target (frt) sites was inserted into 493-bp upstream of exon 11, and the 34-bp loxP sequence into 243-bp downstream of exon 11. Targeting vector pTVC1V2 contained exon 11 of the *Erc1* (*Elks*) gene flanked by loxP sequences, neo gene flanked by two frt, the 3.3-kb upstream and 5.5-kb downstream genomic sequences, and 4.3-kb pMC1DTpA. The targeting vector pTVC1V2 was linearized by SalI and electroporated into embryonic stem (ES) cell line RENKA derived from C57BL/6 strain (Mishina and Sakimura, 2007) as described previously (Takeuchi et al., 2005). G-418 (175 μg/ml)-resistant clones were picked, and recombinant clones were identified by Southern blot hybridization analysis of EcoRV-digested genomic DNA using PCR-amplified 750-bp fragment, PCR-amplified 603-bp fragment, and the 0.6-kb PstI fragment from pLFNeo (Takeuchi et al., 2002) as 5′, 3′, and neo probes, respectively. The neo gene was removed *in vivo* by crossing FLP66 mice carrying the Flp recombinase gene under the control of the *EF1a* promoter. Genotyping of *ELKS* flox mice by PCR was carried out using following primers; 5^′^-AAGGCC CAAACAGAAGTTGA-3^′^ (*ELKS* flox F) and 5^′^-ATGATTTGCTTTCCCATGCT-3^′^ (*ELKS* flox R).

Generation of CASTKO/ELKS fl/fl mice was done by crossbreeding CAST knockout animals (Kobayashi et al., 2016) with ELKS fl/fl to homozygosity.

#### Helper Dependent Adenoviral Cre virus

Helper Dependent Adenoviral viral vectors (HdAd) expressing Cre recombinase were produced as previously described (Lübbert et al., 2017). Briefly, the Cre recombinase expression cassette was cloned into the AscI site of a of pdelta28E4 that has been modified to also contain a separate neurospecific EGFP or myristolated EGFP (mEGFP) expression cassette that is driven by the 470 bp hsyn promoter and the final HdAd plasmid allows for expression of Cre independently of EGFP. Subsequently, the pHAD plasmid was linearized by *PmeI* to expose the ends of the 5′ and 3′ inverted terminal repeats (ITRs) and then transfected into 116 producer cells (Profection Mammalian Transfection System, Promega, Madison, WI, USA). For HdAd production, Helper virus (HV) was added the following day. Forty-eight hours post infection, after cytopathic effects have taken place, cells were subjected to 3 freeze/thaw cycles for lysis and release of the viral particles. To increase the HdAd titer, this lysate was amplified in a total of five serial coinfections of HdAd and HV from 3 × 6 cm tissue culture dishes followed by a 15 cm dish and finally 30 × 15 cm dishes of 116 cells (confluence ~90%, respectively). HdAd was purified by CsCl ultracentrifugation. HdAd was stored at −80°C in storage buffer (10 mM HEPES, 1 mM MgCl_2_, 250 mM sucrose, pH 7.4).

### METHOD DETAILS

#### Stereotaxic injections

Surgery was performed as described previously (Chen et al., 2013; Montesinos et al., 2015). Briefly, at postnatal day 1 (P1) mice were anesthetized by hypothermia, using ice bath for 5 min. Then a total of 1.5 μL HDAd expressing codon optimized Cre recombinase and EGFP (1×10^7^ transducing units (TU)/l) was injected into the cochlear nucleus (CN). Animals were then placed under a warm lamp at 37°C for recovery. After fully recovered, the animals were returned to their respective cages.

#### Immunohistochemistry

Mice were anesthetized with an i.p. injection of Tribromoethanol (250 mg/Kg body weight) and transcardially perfused with ice-cold 0.1 M phosphate buffer (PB) (pH 7.4). Brains were removed and rapidly frozen in OCT using dry ice and 100% ethanol. 5 μm-thick coronal sections of the brainstem were obtained using a cryostat, thaw mounted on microscope slides (Fisherbrand Superfros Plus, Fisher Scientific) and air-dried for 15 min. Sections were fixed by immersion in ice-cold 95% ethanol at 4°C for 30 min followed by acetone for 1 min at RT. Prior to the staining, sections were washed in 0.1 M PB and incubated 20 min at RT in 0.1 M PB blocking solution containing 2% normal goat serum (NGS) and 0.2% Triton X-100. Afterward, sections were incubated at 4°C overnight with the primary antibodies diluted in the same blocking solution (1:100, Rabbit anti-CAST, and 1:100 Rabbit anti-ELKS, T99; 1:100, polyclonal Guinea pig anti-Vglut1, Synaptic Systems). Subsequently, sections were rinsed 3×10 min in 0.1 M PB and incubated with the secondary antibodies diluted in 0.1 M PB containing 0.1% Triton X-100 for 2h at RT 1:200 (Cy 2 AffiniPure goat anti-rabbit IgG (H+L), Jackson Immunoresearch; 1:200, Alexa Fluor® 647-conjugated AffiniPure donkey anti-guinea pig, Jackson Immunoresearch). After 3×10 min in 0.1 M PB washed sections were mounted using Fluoromount-G® mounting medium (SouthernBiotech).

#### Electron microscopy

##### Preembedding immuno-electron microscopy

Wild-type (C57/BL6J) and Cre virus injected *Cast*^−/−^/*Elks*^fl/fl^ P18 mice were anesthetized with Tribromoethanol (250 mg/kg of body weight, i.p.) and perfused transcardially with phosphate-buffered saline (PBS, 150 mM NaCl, 25 mM Sørensen’s phosphate buffer, pH 7.4) followed by fixative solution for 7–9 min containing 4% paraformaldehyde (PFA), 0.5% glutaraldehyde, and 0.2% picric acid in 100 mM Sørensen’s phosphate buffer (PB, pH 7.4). Brains were post-fixed with 4% PFA in PB overnight and 50 μm coronal sections of the brainstem were obtained on a vibratome (Leica VT1200). Expression of EGFP at calyx of Held terminals was visualized using an epifluorescence inverted microscope (CKX41, Olympus) equipped with an XCite Series 120Q lamp (Excelitas technologies), and only those samples showing EGFP were further processed as previously reported (Montesinos et al., 2015). After washing with PB, sections were cryoprotected with 10%, 20% and 30% sucrose in PB and submersed into liquid nitrogen, then thawed. Then sections were incubated in a blocking solution containing 10% normal goat serum (NGS), 1% fish skin gelatin (FSG), in 50 mM Tris-buffered saline (TBS, 150 mM NaCl, 50 mM Tris, pH 7.4) for 1h, and incubated with an anti-GFP antibody (0.1μg/ml, ab6556, Abcam) diluted in TBS containing 1% NGS, 0.1% FSG plus 0.05% NaN_3_ at 4°C for 48h. After washing with TBS, sections were incubated overnight in nanogold conjugated goat anti-rabbit IgG (1:100, Nanoprobes) diluted in TBS containing 1% NGS and 0.1% FSG. Immunogold-labeled sections were washed in PBS, briefly fixed with 1% glutaraldehyde in PBS, and silver intensified using HQ silver intensification kit (Nanoprobe). After washing with PB, they were treated with 0.5% OsO_4_ in 0.1 M PB for 20 min, en-bloc stained with 1% uranyl acetate, dehydrated and flat embedded in Durcupan resin (Sigma-Aldrich). After trimming out the MNTB region, ultrathin sections were prepared with 40 nm-thickness using an ultramicrotome (EM UC7, Leica). Sections were counterstained with uranyl acetate and lead citrate, and examined in a Tecnai G2 Spirit BioTwin transmission electron microscope (ThermoFisher, formerly FEI) at 100kV acceleration voltage. Images were taken with a Veleta CCD camera (Olympus) operated by TIA software (ThermoFisher, formerly FEI). Images used for quantification were taken at 60,000x magnification.

##### SDS-digested freeze fracture replica labeling

Mice were injected with Cre virus harboring myristoylated EGFP at P1. P18 animals were anesthetized with Tribromoethanol (250 mg/kg of body weight, i.p.) and perfused transcardially with PBS followed by 2% PFA, and 0.2% picric acid in 0.1 M PB for 12 min. 130 μm-thick coronal sections of brainstem were obtained using a vibratome (Leica VT1200). Only those samples showing myristoylated EGFP expression were selected. The brain slices containing the MNTB region were cryoprotected in 10%, 20% and 30% glycerol at 4°C overnight. Small pieces containing MNTB region were trimmed out and frozen using a high pressure freezing machine (HPM100, Leica). Frozen samples were fractured in two halves using a double replication device at −120°C, replicated first with a 2 nm carbon deposition, shadowed from 60 degree angle by carbon-platinum of 2.0 nm and supported by a final carbon deposition of 20–30nm, using a JFDV Freeze fracture machine (JEOL/Boeckeler). The tissue was dissolved by placing the replicas in a digesting solution containing 2.5% SDS, 20% sucrose and 15 mM Tris-HCl (pH 8.3) with gentle agitation in an oven at 82.5°C for 18 hr. The replicas were washed and blocked with 4% BSA and 1% FSG in TBS for 1 hr, then incubated with a mixture of primary antibody; rabbit anti- GFP (1 μg/ml, AbCam, cat# ab6556) and guinea pig anti-alpha 1A subunit of Ca_V_2.1 (0.7 μg/ml, Synaptic Systems, cat# 152205) diluted in 0.04% BSA and 0.01% FSG in TBS for 18 h at room temperature. After several washes, the replicas were incubated in donkey anti-rabbit IgG conjugated to 6 nm gold particles and donkey anti-guinea pig IgG conjugated to 12 nm gold particles (1:30 respectively; Jackson Immunoresearch) diluted in 0.04% BSA and 0.01% FSG in TBS for 18 h at room temperature. After being washed, the replicas were picked up on 100-parallel-bar copper grids or aperture grids and examined with a Tecnai G2 Spirit BioTwin transmission electron microscope (ThermoFisher, formerly FEI) at 100 kV acceleration voltage. Images were taken with a Veleta CCD camera (Olympus).

#### Electrophysiology

All recordings were carried out with a HEKA EPC 10/2 amplifier controlled by Patch-Master (Harvard Apparatus). Current responses were sampled in 20 μs intervals and low-pass filtered at 6 kHz. To identify calyces transduced with the HdAd expressing Cre recombinase and EGFP, the slice was illuminated with a Polychrome V (ThermoFisher, formerly FEI) xenon bulb monochromator using a 480 nm wavelength. Postsynaptic patch pipettes were filled with the following solution (in mM): 145 Cs-gluconate, 20 TEA-Cl, 10 HEPES, 5 Na2-phosphocreatine, 4 MgATP, 0.3 NaGTP, 6 QX-314, and 5 EGTA. All recordings were performed at room temperature (≈25°C).

##### Slice preparation

Acute brain slices were prepared as previously described (Chen et al., 2013). Briefly, the mice were decapitated and the brains were immersed in low calcium artificial cerebrospinal fluid (aCSF) containing (in mM): 125 NaCl, 2.5 KCl, 10 glucose, 25 NaHCO_3_, 1.25 NaH_2_PO_4_, 0.4 L-ascorbic acid, 3 myo-inositol, 2 Na-pyruvate, 3 MgCl_2_ and 0.1 CaCl_2_ continuously bubbled with 95% O_2_-5% CO_2_ (pH ≈7.3). Acute brainstem slices with MNTB were obtained using Leica VT 1200 or Campden 7000smz-2 vibratome (P16-P21, 150–200 μm thick slices). Slices were immediately transferred to an incubation beaker containing normal aCSF (same as the low-calcium aCSF except that 1 mM MgCl_2_ and 1, 1.2 or 2 mM CaCl_2_ were used) at 37°C, continuously bubbled with 95% O_2_-5% CO_2_. The slices were allowed for recovery for 45 min and then were transferred to a recording chamber with the same aCSF solution at room temperature (≈25°C).

##### Afferent fiber stimulation

Afferent fiber stimulation was performed as described previously (Forsythe and Barnes-Davies, 1993a, 1993b). Briefly, a bipolar electrode was placed between brainstem midline and MNTB. MNTB principal neurons were whole-cell voltage clamped at −60 mV. 1.2mM external Ca^2+^ were used for recording on P17-P21 calyces. Evoked EPSCs were recorded with the use of 0.25 mM kynurenic acid (Tocris Bioscience) to minimize receptor saturation (Trussell, 1998); 50 μM D-AP-5 (Tocris Bioscience) to block NMDA receptor, 20 μM bicuculline (Tocris Bioscience) and 5 μM strychnine (Tocris Bioscience) to block IPSCs. Patch pipettes had open tip resistances of 3–4 MΩ and were filled with (in mM): 145 Cs-gluconate, 20 TEA-Cl, 10 HEPES, 5 Na_2_-phosphocreatine, 4 MgATP, 0.3 NaGTP, 6 QX-314, and 5 EGTA. Series resistance (Rs, 3–8 MΩ) were online compensated to 3 MΩ and the remaining Rs was offline compensated to 0 MΩ for all EPSCs (Traynelis, 1998). Experiment with different extracellular Ca^2+^ concentrations was performed using 0.05Hz stimulation frequency while ACSF with different Ca^2+^ concentrations (mM: 0.2, 0.5, 0.75, 1, 1.5) was perfused. In this set of experiments kynurenic acid was omitted and all cells were online Rs compensated till 3 MU and offline correction was not performed.

##### Presynaptic Ca^2+^ current recordings

1 mM external Ca^2+^ was used for all presynaptic recordings to minimize voltage error. 100 μM 4-AP (Tocris Bioscience), 20 mM TEA (Sigma Aldrich) and 1 μM TTX (Alomone Labs) were used to isolate Ca^2+^ currents. Presynaptic patch pipettes were filled with same solution as of postsynaptic patch pipettes except without QX-314 and with 0.5 mM EGTA. Presynaptic terminal was whole-cell voltage clamped at −80 mV and 10 ms step depolarization from −70 mV to 40 mV were applied at 0.1 Hz. Rs was typically < 15 MΩ and online compensated to 6 MΩ. Leak current was < 120 pA.

##### Paired pre- and postsynaptic recordings

External Ca^2+^ concentration was 2 mM for pair recordings. 2 mM kynurenic acid, 100 μM cyclothiazide (Tocris Bioscience), 50 μM D-AP5, 100 μM 4-AP, 20 mM TEA and 1 μM TTX were included in external solution. Presynaptic calyx terminals and postsynaptic principal neurons of MNTB were simultaneously whole-cell voltage-clamped at −80 mV and −60 mV, respectively. Patch pipettes had an open tip resistance of 5–6 MΩ and 3–4 MΩ for presynaptic and postsynaptic recordings, respectively. Presynaptic R_S_ (< 15 MΩ) was online compensated to 8 MΩ. Postsynaptic R_S_ (< 8 MΩ) was online compensated to 3 MΩ, and remaining R_S_ was compensated offline to 0.

##### Miniature postsynaptic currents (mEPSCs)

External Ca^2+^ of 2 mM was used for mEPSCs recording. MNTB principal neurons were whole-cell voltage clamped at −80 mV. aCSF was supplemented with 50 μM D-AP5, 20 μM bicuculline, 5 μM strychnine, 1 μM TTX and 20 mM TEA. Rs (< 8 MΩ) was not compensated. For purpose of testing effects of VGCC on mEPSCs frequency 200 μM Cd^2+^ was added to the recording solution. Prior to that, several minutes of recordings were done in order to establish a baseline mEPSC frequency for each cell. First 2 min upon application of Cd^2+^ were not used for analysis to assure that drug is effectively present in the slice.

#### Resting free Ca^2+^ imaging at the presynaptic terminal

Experiments were performed as previously described (Di Guilmi et al., 2014; Kochubey and Schneggenburger, 2011). Briefly, we used an external Ca^2+^ concentration of 2 mM. Presynaptic terminals were whole-cell voltage-clamped for 2–3 min at −80 mV with pipettes containing (in mM: 125 K-gluconate, 20 TEA-Cl, 20 HEPES, 5 Na-ATP, 0.3 Na_2_-GTP, 0.1 Fura-2, pH = 7.2). The setup for imaging contained a monochromator (Polychrome V, ThermoFisher) exciting Fura-2 at 340 and 380 nm and an EMCCD camera (Luca, Andor Technology, Belfast, UK) controlled by LifeAcquisition software (ThermoFisher).

### QUANTIFICATION AND STATISTICAL ANALYSIS

#### Confocal imaging and image analysis

Confocal single images were acquired with a Zeiss LSM 780 Confocal Scanning Microscope. Confocal scans for each fluorochrome was acquired sequentially using 63x/1.3(NA) Apochromat MultiImmersion objective. The intensity emission signal from each channel was adjusted to below saturation level. Images were processed with Fiji imaging analysis software (http://fiji.sc/).

#### TEM image analysis

We identified calyces which were positive for Cre expression by immunogold labeling with an anti-GFP antibody, and compared EGFP-positive terminals (*Cast*^−/−^/*Elks*^−/−^) to EGFP-negative terminals (*Cast*^−/−^) in the same slice or to calyces in the wild-type sample. All TEM data were analyzed using Fiji imaging analysis software (http://fiji.sc/). Each presynaptic active zone (AZ) was defined as the membrane directly opposing postsynaptic density, and the length of each AZ was measured. Vesicles within 200 nm from each AZ were manually selected and their distances relative to the AZ were calculated using a 32-bit Euclidean distance map generated from the AZ. For data analysis, vesicle distances were binned every 5 nm and counted (Montesinos et al., 2015; Taschenberger et al., 2002). Vesicles less than 5 nm from the AZ were considered “docked.” Three animals for each condition and 40 AZs per animal were analyzed.

#### SDS-FFRL image collection and analysis

Presynaptic P-faces (protoplasmic face of the cell membrane) of the calyx of Held were morphologically identified by existence of cross-fractured cytoplasm containing synaptic vesicles and/or by existence of glutamate receptor clusters uniquely shown on the adjacent E-face (exoplasmic face) of the postsynaptic cell of principle neuron of MNTB (Budisantoso et al., 2012; Nakamura et al., 2015). Specifically, immunogold-labeled presynaptic P-faces that were in contact with the postsynaptic soma were imaged at 43,000x magnification. EGFP-positive presynaptic P-faces were identified by the existence of 6-nm immunogold particles as *Cast*^−/−^/*Elks*^−/−^, and EGFP negative calyx terminals were categorized as *Cast*^−/−^. We analyzed the distribution and number of Ca_V_2.1 from the 12 nm immunogold labeling in both EGFP positive and negative terminals. Eight P-faces from the replicas of *Cast*^−/−^ and *Cast*^−/−^/*Elks*^−/−^ samples (16 total), including small to large pieces, were analyzed comprising 102.8 and 83.9 μm^2^ of P-face area, respectively. For each continuous P-face, images were manually stitched and minimally adjusted for brightness and contrast (Adobe Photoshop CS6), and 12 nm gold particles corresponding to Ca_V_2.1 channels were thresholded and quantified using Microscopy Image Browser (MIB) (Belevich et al., 2016). A custom macro was used in Fiji to extend a circle with a 30 and 100 nm radius from each large gold particle to form clusters of closely distributed Ca_V_2.1 channels (Althof et al., 2015; Nakamura et al., 2015). Clusters and the particles within them were then analyzed.

#### Electrophysiological data analysis

Electrophysiological data were analyzed offline with Fitmaster (Heka) and custom programming written in Igor Pro (version 6.36; Wavemetrics).

Peak Ca^2+^ current-voltage relationships were fitted according to a Hodgkin-Huxley formalism assuming four independent gates and Goldman-Hodgkin-Katz for open-channel conductance G:

$$I(V)=\Gamma *\frac{1-e^{-\frac{V-E_{rev}}{25\;mV}}}{1-e^{-\frac{V}{25\;mV}}}*{\left(1-e^{\frac{V-V_{m}}{k_{m}}}\right)}^{-4}$$

with E_rev_ as reversal potential, V_m_ as half-maximal activation voltage per gate, and k_m_ as the voltage-dependence of activation. Tail currents were measured as peaks minus baseline, plotted as a function of voltage and fitted with a Boltzmann function:

$$I_{tail}(\text{V})=I_{\mathrm{base}}+\frac{I_{min}}{1+{\text{e}}^{-\frac{V-V_{1/2}}{k}}}$$

where V_1/2_ represents the half-maximal voltage and k the corresponding slope factor.

EPSC amplitudes were measured as peak minus baseline. For determining RRP size a stimulation frequency of and 300 Hz was used and back extrapolation method was implemented (Neher, 2015).

Data for the different external Ca^2+^ concentrations were fitted with sigmoid function:

$$f(x)=\frac{max}{\left(1+e^{\frac{x_{half}-x}{rate}}\right)}$$

With *max* as indicated level of saturation, *x*_*half*_ as symmetric inflection point and *rate* indicated slope.

For analysis of mEPSCs custom written script by H. Taschenberger in Igor Pro was used. In experiment where Cd^2+^ was added in extracellular solution (Figure 5D) moving average of 5 s was used for determining frequency of mEPSCs.

#### Analysis of ratiometric Ca^2+^ imaging

We took a series of 20 images (F340/F380) with an exposure time of 100 ms per image and analyzed the data in ImageJ. Fluorescence intensities were quantified after background subtraction. The Fura-2 calibration curve was based on 10 individual free Ca^2+^ concentrations buffered with EGTA from Ca^2+^-free up to 1.4 μM mixed with K-gluconate internal solution in 50 μm rectangle glass cuvettes (Vitrocom) (Kochubey and Schneggenburger, 2015). The ratios obtained at the presynaptic terminal were translated into free Ca^2+^ concentrations using the constants R_min_, R_max_, K_eff_ (disscociation constant was 239.95 nM at 25°C and pH = 7.2) according to this equation (Grynkiewicz et al., 1985):]

$$\left[{\mathrm{Ca}}^{2+}\right]=K_{eff}*\frac{R-R_{min}}{R_{max}-R}$$

#### Statistics

Statistics were performed with Prism7 (GraphPad Software). A Shapiro-Wilk test was used to check for normal distribution of respective data group and Bartlett’s test to check for comparable variance among groups. To compare two normally distributed groups, an unpaired two-tailed Student’s t test was used. To compare more than two normally distributed groups, a one-way ANOVA with a post hoc Dunnett’s test was used. To compare not normally distributed groups a Kruskal-Wallis with a post hoc Dunn’s test was used. For comparison of binned values in Figure 2E, Fisher’s exact test was used. For comparing the before and after drug application (Figure 5E) paired t test was used. Data are reported as mean ± SEM. Statistical significance was accepted at p < 0.05.

## Supplementary Material

1

## Figures and Tables

**KEY RESOURCES TABLE:** 

REAGENT or RESOURCE	SOURCE	IDENTIFIER
Antibodies		

Rabbit polyclonal anti-CAST	Dr Ohtsuka, University of Yamanashi, Japan	N/A
Rabbit polyclonal anti-ELKS	Dr Ohtsuka, University of Yamanashi, Japan	N/A
Guinea pig polyclonal anti vGlut1	Synaptic Systems	Cat# 135 304, RRID: AB_887878
Cy2 Goat anti-rabbit	Jackson IR	Cat# 111–225–144, RRID: AB_2338021
Alexa Fluor 647 donkey anti-guinea pig	Jackson IR	Cat# 706–605–148, RRID: AB_2340476
Rabbit anti- GFP	Abcam	Cat# ab6556, RRID: AB_305564
Guinea pig anti-alpha 1A	Synaptic systems	Cat# 152205, RRID: AB_2619842
Donkey anti rabbit IgG	Jackson IR	Cat# 711005152, RRID: AB_2340585
12 nm Colloidal Gold-AffiniPure Donkey anti Guinea pig IgG	Jackson IR	Cat# 706205148, RRID: AB_2340465
6 nm Colloidal Gold-AffiniPure Donkey anti Guinea pig IgG	Jackson IR	Cat# 711195152 RRID: AB_2340585

Bacterial and Virus Strains		

HdAd 28E4 syn iCre EGFP	Dr Young Laboratory, University of Iowa	N/A
HdAd 28E4 syn iCre mEGFP	Dr Young Laboratory, University of Iowa	N/A
AAV9-CMV-NlsCre-WPRE	Dr Inoue at Kyoto University	N/A

Chemicals, Peptides, and Recombinant Proteins		

Kynurenic acid	Tocris Bioscience	Cat# 0223
Qx-314	Tocris Bioscience	Cat# 2313
D-AP5	Tocris Bioscience	Cat# 0106
BICUCULLINE	Tocris Bioscience	Cat# 0131
STRYCHNINE	Tocris Bioscience	Cat# 2785
4-aminopyridine - 4-AP	Tocris Bioscience	Cat# 0940
Tethraethylamonium chloride -TEA	Sigma Aldrich	Cat# T-2265
Tetrodotoxin -TTX	Alomone labs	Cat# T-550
Fura-2	TEFLabs	Cat# 0104

Experimental Models: Organisms/Strains		

Mice: C57/BL6J	Jackson Laboratory	000664
Mice: *Cast*^−/−^, *Elks^fl/fl^*	Dr Ohtsuka, University of Yamanashi, Japan	N/A
Mice: *Cast*^+/+^, *Elks^fl/fl^*	Dr Ohtsuka, University of Yamanashi, Japan	N/A

Oligonucleotides		

Primer: Elks F - 5′-AAGGCCCAAACAGAAGTTGA-3′	This study	N/A
Primer: Elks R - 5′-ATGATTTGCTTTCCCATGCT-3′	This study	N/A
Primer: CAST WT F - 5′-GTCACCACGTCTGCCAAGGT-3′	This study	N/A
Primer: CAST KO F - 5′-GACATAGCGTTGGCTACCCGT-3′	This study	N/A
Primer: CAST KO R -5′-GGGCTTGAAGATCCAACATCG-3′	This study	N/A

Recombinant DNA		

RP24-103F1 BAC clone	BACPAC resource center	RP24-103F1

Software and Algorithms		

Fiji	N/A	http://fiji.sc/
Patch Master	Harvard Apparatus	http://www.heka.com/downloads/downloads_main.html
Igor Pro 6.37	Wavemetrics	https://www.wavemetrics.com/
Prism	Graphpad	https://www.graphpad.com/scientific-software/prism/

## Highlights

- Loss of CAST/ELKS reduces presynaptic Ca_V_2.1 currents and channels
- Deletion of CAST/ELKS increases *Pr*, spontaneous release, while reducing RRP size
- CAST/ELKS are positive regulators of Ca_V_2.1 channel abundance
- CAST/ELKS regulate multiple steps in the synaptic vesicle life cycle
