# Supplementary material for: CAST/ELKS Proteins Control Voltage-Gated Ca2+ Channel Density and Synaptic Release Probability at a Mammalian Central Synapse
Source: Cell Rep. Author manuscript; Available in PMC 2019 Feb 12. (PMC6372087; doi:10.1016/j.celrep.2018.06.024)
Supplement: 1 [file NIHMS1000840-supplement-1.pdf]

Cell Reports, Volume *24*

Supplemental Information

CAST/ELKS Proteins Control Voltage-Gated Ca^{2+} Channel Density and Synaptic Release Probability at a Mammalian Central Synapse

Wei Dong, Tamara Radulovic, R. Oliver Goral, Connon Thomas, Monica Suarez Montesinos, Debbie Guerrero-Given, Akari Hagiwara, Travis Putzke, Yamato Hida, Manabu Abe, Kenji Sakimura, Naomi Kamasawa, Toshihisa Ohtsuka, and Samuel M. Young Jr.

Supplemental Information

Supplemental data

Figure S1. Targeting strategy of ELKS KO mice.

Figure S2. Depletion of ELKS following to the expression of Cre recombinase was examined using primary culture pyramidal neurons.

Figure S3. Loss of ELKS protein did not affect AP-evoked release.

Figure S4. The loss of CAST/ELKS does not change Ca^{2+} sensitivity of basal AP evoked release.

Table S1 (related to Figure 1).

Table S2 (related to Figure 2 and 4).

Table S3 (related to Figure 3).

Table S4 (related to Figure 4).

Table S5 (related to Figure 5).

SUPPLEMENTAL DATA

Figure S1

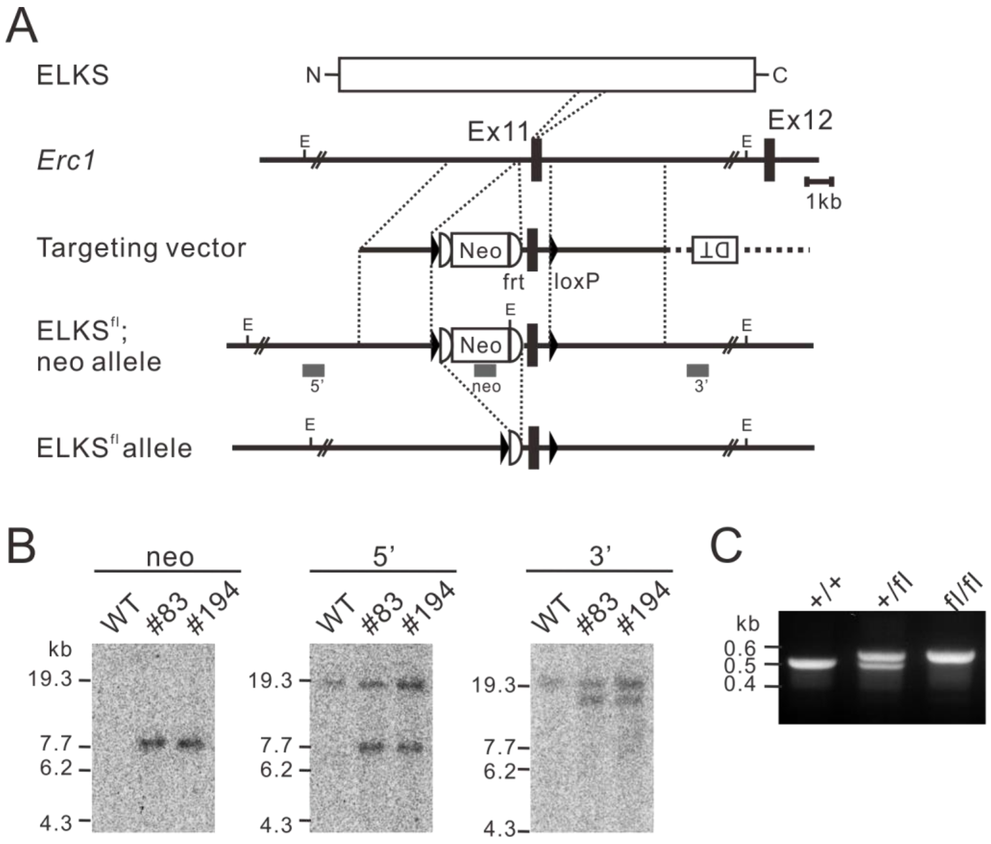

Figure S1. Targeting strategy of ELKS KO mice. (Related to STAR Methods, Generation of *Elks* flox mice) Schematic representation of ELKS, *ELKS*/*Erc1* genomic DNA, targeting vector, the floxed and neo-inserted allele (*ELKS^{flox}; neo* allele), and the floxed allele (*ELKS^{flox}* allele). The *ELKS^{flox}; neo* allele contains two loxP sequences flanking exon 11 of the *ELKS* gene and the *neo* gene flanked by two *frt* sequences. The *neo* gene was removed by crossing FLP66 mice carrying the Flp recombinase. DT, diphtheria toxin gene; E, EcoRV; Neo, neomycin resistant gene; fl, flox. (B) Southern blot analysis of EcoRV-digested genomic DNA of wild type (WT) and heterozygous ES cells (clones 83 and 194). The *ELKS* flox line was established from clone 83. (C) Genomic PCR analysis of WT (+/+), and the *ELKS^{flox} neo* allele (+/flox, flox/flox) in A.

Figure S2

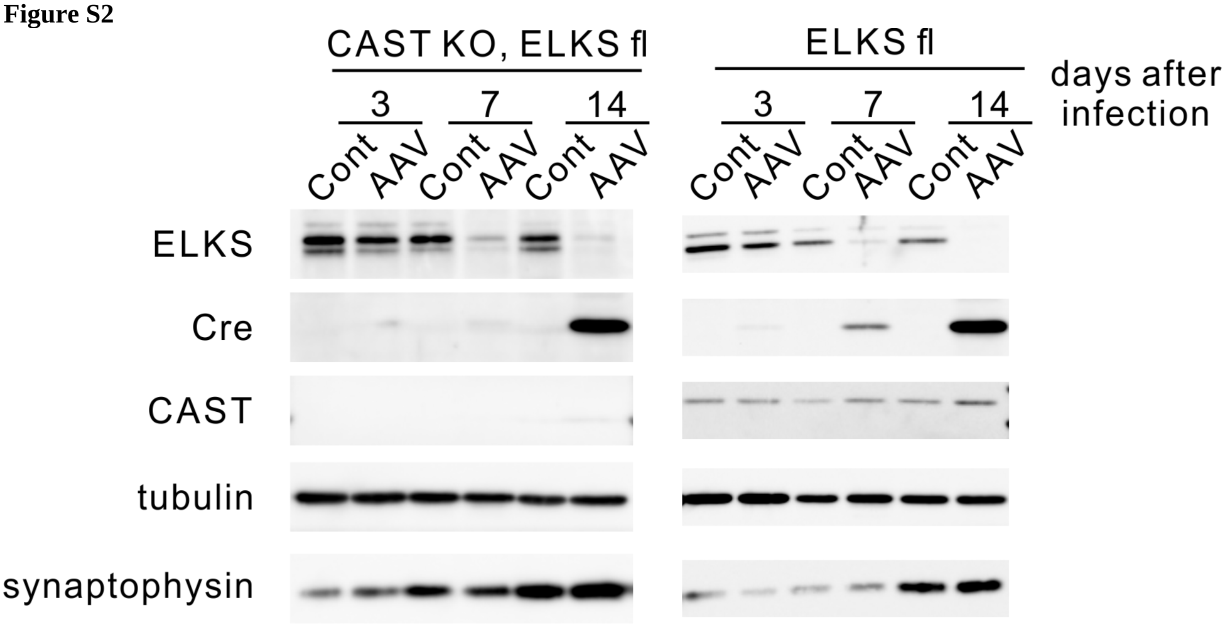

Figure S2. **Depletion of ELKS following to the expression of Cre recombinase was examined using primary culture pyramidal neurons. (Related to STAR Methods, Generation of *Elks* flox mice)**
Cortical neuron cultures were prepared from postnatal day 0 (P0) ELKS fl or CAST KO; ELKS fl pups. Neurons were further treated with AAV9-CMV-NlsCre-WPRE (a kind gift from Dr. Inoue at Kyoto University) at 7 DIV, and samples were collected 3 (10DIV), 7 (14DIV), and 14 (21DIV) days after. Lysate of AAV treated and non-treated (Cont) samples were examined by western blotting with indicated antibodies. As Cre recombinase expressed 7 and 14 days after infection, ELKS was depleted. The expression of CAST was completely abolished in CAST KO; ELKS fl neurons. The synaptic vesicle marker, synaptophysin showed maturation of culture neuron synapses.

Figure S3

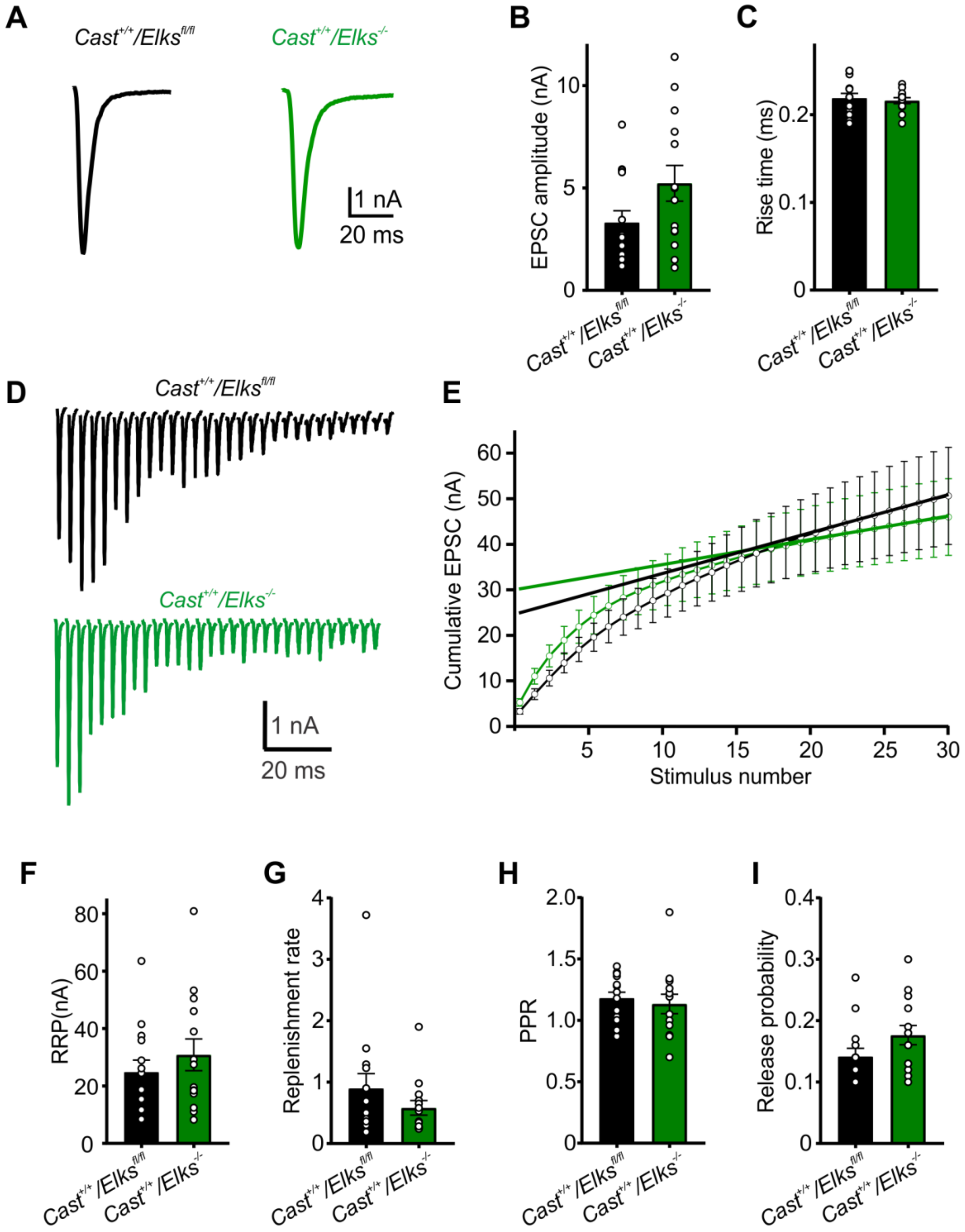

Figure S3. Loss of ELKS does not affect AP-evoked release. (Related to Figure 3)
(A) Representative EPSC evoked with single AP using afferent fiber stimulation. (B, C) Mean EPSC amplitude and rise times. (D) Representative train of EPSCs evoked by 30 APs at 300 Hz from *Cast*$^{+/+}$*/Elks*$^{fl/fl}$ and *Cast*$^{+/+}$*/Elks*$^{-/-}$ (top and bottom respectively). (E) Mean cumulative EPSCs plot against stimulus number. (F, G, H, I) Mean values

for size of RRP, replenishment rate, PPR and release probability. (n = 14 for both group, *t-test* or Mann-Whitney test, data are represented as mean ± SEM.)

Figure S4

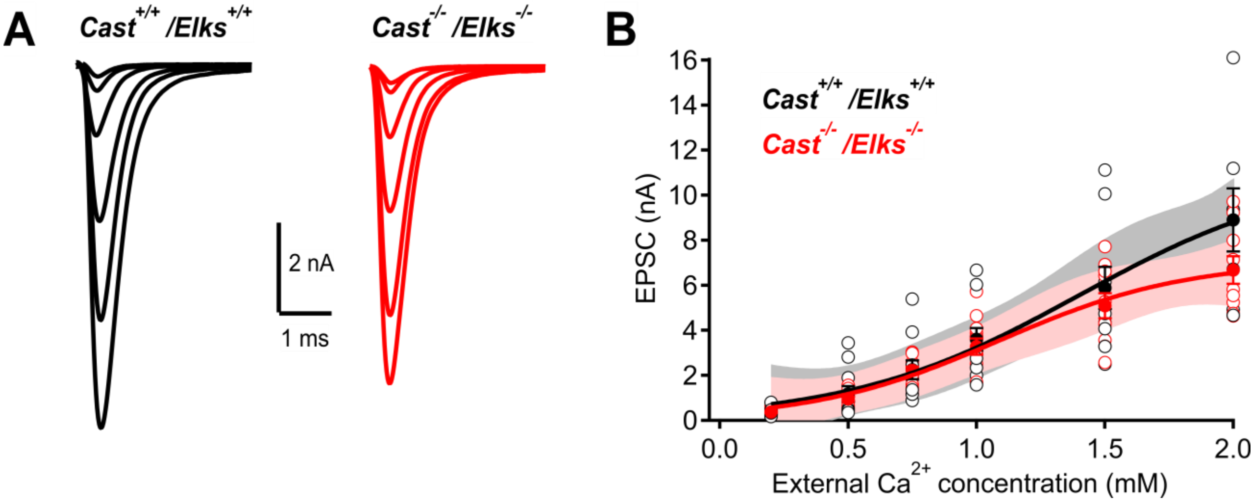

Figure S4. The loss of CAST/ELKS does not change Ca^{2+} sensitivity of basal AP evoked release. (Related to Results, Loss of CAST/ELKS proteins and their impact on Ca^{2+} sensitivity of basal AP evoked transmission release.)

(A) Mean traces of basal EPSCs evoked at 0.05 Hz. Each trace presents mean for different external Ca^{2+} concentration (mM): 0.2, 0.5, 0.75, 1, 1.5, 2. (B) EPSCs amplitudes recorded in respective external Ca^{2+} concentrations; open circles individual cells, closed circles mean with standard errors; grey and light red filling correspond to 95% confidence intervals for *Cast*$^{+/+}$ *Elks*$^{+/+}$ and *Cast*$^{-/-}$*Elks*$^{-/-}$ respectively (mean ± SEM (n) for mM: 0.2, 0.5, 0.75, 1, 1.5, 2, *Cast*$^{+/+}$/*Elks*$^{+/+}$: 0.33 ± 0.06 (10), 1.18 ± 0.34 (10), 2.24 ± 0.42 (10), 3.59 ± 0.5 (10), 5.87 ± 0.93 (9), 8.89 ± 1.4 (7); *Cast*$^{-/-}$/*Elks*$^{-/-}$: 0.37 ± 0.04 (12), 0.95 ± 0.1 (12), 2.22 ± 0.17 (11), 3.27± 0.36 (12), 5.09 ± 0.55 (10), 6.67± 0.61 (9), respectively).

Table S1. Related to Figure 1, presynaptic recordings summary values.

Parameter (unit)	Mean	SEM	n	P	statistics
**max. I_{Ca} (pA)**					
Cast$^{+/+}$/*Elks*$^{+/+}$	873	63	10	control	One Way
Cast$^{-/-}$/*Elks*$^{+/+}$	953	67	12	0.8547 (ns)	ANOVA
Cast$^{-/-}$/*Elks*$^{-/-}$	499	48	10	0.0002 (***)	Dunnett's test
**Membrane capacitance C_{slow} (pF)**					
Cast$^{+/+}$/*Elks*$^{+/+}$	19.7	1.1	10	control	One Way
Cast$^{-/-}$/*Elks*$^{+/+}$	17.0	1.1	12	0.2311 (ns)	ANOVA
Cast$^{-/-}$/*Elks*$^{-/-}$	19.5	1.6	10	0.9957 (ns)	Dunnett's test
**Ca^{2+} currents as function of voltage**					
**Half-maximal activation voltage V_m (mV)**					
Cast$^{+/+}$/*Elks*$^{+/+}$	-29.0	1.5	10	control	One Way
Cast$^{-/-}$/*Elks*$^{+/+}$	-26.1	0.8	12	0.1528 (ns)	ANOVA
Cast$^{-/-}$/*Elks*$^{-/-}$	-22.5	1.1	10	0.0007 (***)	Dunnett's test
**Voltage-dependence of activation k_m (mV)**					
Cast$^{+/+}$/*Elks*$^{+/+}$	6.3	0.4	10	control	One Way
Cast$^{-/-}$/*Elks*$^{+/+}$	6.2	0.3	12	0.8912 (ns)	ANOVA
Cast$^{-/-}$/*Elks*$^{-/-}$	8.3	0.4	10	0.0014 (**)	Dunnett's test
**Ca^{2+} tail currents as function of voltage**					
**Half-maximal activation voltage $V_{0.5}$ (mV)**					
Cast$^{+/+}$/*Elks*$^{+/+}$	-18.7	1.9	10	control	One Way
Cast$^{-/-}$/*Elks*$^{+/+}$	-15.5	1.0	12	0.375 (ns)	ANOVA
Cast$^{-/-}$/*Elks*$^{-/-}$	-10.2	2.1	10	0.0025 (**)	Dunnett's test
**Voltage-dependence of activation k (mV)**					
Cast$^{+/+}$/*Elks*$^{+/+}$	6.2	0.5	10	control	One Way
Cast$^{-/-}$/*Elks*$^{+/+}$	6.2	0.3	12	0.9822 (ns)	ANOVA
Cast$^{-/-}$/*Elks*$^{-/-}$	7.2	0.5	10	0.2353 (ns)	Dunnett's test

Table S2. Related to Figure 2 and Figure 4, EM and SDS-FFRL summary values.

**Parameter (unit)**	**Mean**			
**Total area analyzed (µm^2)**				
Cast$^{-/-}$/Elks$^{+/+}$	83.9			
Cast$^{-/-}$/Elks$^{-/-}$	102.8			
**Total # of gold particles**				
Cast$^{-/-}$/Elks$^{+/+}$	2591			
Cast$^{-/-}$/Elks$^{-/-}$	1726			
**Total # of clusters**				
Cast$^{-/-}$/Elks$^{+/+}$	441			
Cast$^{-/-}$/Elks$^{-/-}$	330			
**Number of single particles**	**Total**			
Cast$^{-/-}$/Elks$^{+/+}$	168			
Cast$^{-/-}$/Elks$^{-/-}$	181			
**Parameter (unit)**	**Mean [median]**	**SEM**	**P**	**statistics**
**Gold particles per cluster**				
Cast$^{-/-}$/Elks$^{+/+}$	5.5 [4]	0.22	control	Mann-Whitney
Cast$^{-/-}$/Elks$^{-/-}$	4.7 [3]	0.21	<0.05(*)	U test
**Cluster area (µm^2)**				
Cast$^{-/-}$/Elks$^{+/+}$	0.0097 [0.0074]	0.0003	control	Mann-Whitney
Cast$^{-/-}$/Elks$^{-/-}$	0.0086 [0.0066]	0.0003	<0.05 (*)	U test
**Gold particle density (#/µm^2 of cluster area)**				
Cast$^{-/-}$/Elks$^{+/+}$	541.2 [523.4]	5.2	control	Mann-Whitney
Cast$^{-/-}$/Elks$^{-/-}$	516.1 [495.1]	5.5	<0.01 (**)	U test
**Putative AZ area (µm^2)**				
Cast$^{-/-}$/Elks$^{+/+}$	0.0964 [0.078]	0.0043	control	Mann-Whitney
Cast$^{-/-}$/Elks$^{-/-}$	0.0725 [0.0647]	0.0023	<0.001(***)	U test
**Cluster number per putative AZ**				
Cast$^{-/-}$/Elks$^{+/+}$	1.6 [1]	0.09	control	Mann-Whitney
Cast$^{-/-}$/Elks$^{-/-}$	1.07 [1]	0.05	<0.0001 (****)	U test

Parameter (unit)	Mean [median]	SEM	P	statistics
**AZ length (nm)**				
Cast$^{+/+}$/*Elks*$^{+/+}$	283 [260]	7	control	Mann-Whitney
Cast$^{-/-}$/*Elks*$^{-/-}$	277 [269]	7	ns	U test
**Number of docked SV**				
Cast$^{+/+}$/*Elks*$^{+/+}$	1.5 [1]	0.1	control	Mann-Whitney
Cast$^{-/-}$/*Elks*$^{-/-}$	1.4 [1]	0.1	ns	U test

***AZ- active zone; SV- synaptic vesicle.**

Table S3. Related to Figure 3. Afferent fiber stimulation data.

Parameter (unit)	Mean	SEM	n	P	statistics
**RRP (nA)**					
Cast$^{+/+}$/*Elks*$^{+/+}$	45.59	6.3	15	control	Kruskal-Wallis
Cast$^{-/-}$/*Elks*$^{+/+}$	49.06	6.11	15	>0.999 (ns)	
Cast$^{-/-}$/*Elks*$^{-/-}$	19.82	3.12	16	0.0022 (**)	Dunn's test
**PPR**					
Cast$^{+/+}$/*Elks*$^{+/+}$	1.45	0.05	15	control	Kruskal-Wallis
Cast$^{-/-}$/*Elks*$^{+/+}$	1.47	0.38	15	>0.999 (ns)	
Cast$^{-/-}$/*Elks*$^{-/-}$	0.96	0.05	16	0.0004 (***)	Dunn's test
**Pr**					
Cast$^{+/+}$/*Elks*$^{+/+}$	0.12	0.008	15	control	One-Way
Cast$^{-/-}$/*Elks*$^{+/+}$	0.135	0.012	15	0.58 (ns)	ANOVA
Cast$^{-/-}$/*Elks*$^{-/-}$	0.204	0.012	16	0.0001(****)	Dunnett's test
**EPSC (nA)**					
Cast$^{+/+}$/*Elks*$^{+/+}$	5.22	0.6	15	control	One-Way
Cast$^{-/-}$/*Elks*$^{+/+}$	5.98	0.66	15	0.61 (ns)	ANOVA
Cast$^{-/-}$/*Elks*$^{-/-}$	4.06	0.62	16	0.32 (ns)	Dunnett's test
**Replenishment rate**					
Cast$^{+/+}$/*Elks*$^{+/+}$	0.78	0.11	15	control	Kruskal-Wallis
Cast$^{-/-}$/*Elks*$^{+/+}$	0.76	0.08	15	>0.999 (ns)	
Cast$^{-/-}$/*Elks*$^{-/-}$	0.47	0.05	16	0.044 (*)	Dunn's test
**Basal EPSC amplitude**					
Cast$^{+/+}$/*Elks*$^{+/+}$	5.22	0.54	15	control	One-Way
Cast$^{-/-}$/*Elks*$^{+/+}$	6.71	0.7	14	0.23 (ns)	ANOVA
Cast$^{-/-}$/*Elks*$^{-/-}$	4.8	0.8	16	0.87 (ns)	Dunnett's test
**Half width (µs)**					
Cast$^{+/+}$/*Elks*$^{+/+}$	492	16.45	15	control	One-Way
Cast$^{-/-}$/*Elks*$^{+/+}$	452.6	20.09	14	0.22 (ns)	ANOVA
Cast$^{-/-}$/*Elks*$^{-/-}$	491.5	16.73	16	0.9996 (ns)	Dunnett's test

***RRP-readily releasable pool, PPR- paired pulse ratio, Pr-release probability, EPSC-excitatory postsynaptic current.**

Table S4. Related to Figure 4, paired recordings data.

Parameter(unit)	Mean	SEM	(n)	P					
**Capacitance pre C_{slow} (pF)**									
$Cast^{+/+}/Elks^{+/+}$	14.9	0.5	6	control					
$Cast^{-/-}/Elks^{-/-}$	12.7	1.1	5	0.0947 (ns)					
	**1 ms**					**10 ms**			
	**Mean**	**SEM**	**(n)**	**P**	**Stat.**	**Mean**	**SEM**	**(n)**	**P**
**EPSC amplitude (nA)**									
$Cast^{+/+}/Elks^{+/+}$	12.3	1.9	6	control	t-test	12.2	2.1	6	control
$Cast^{-/-}/Elks^{-/-}$	4.6	1.2	5	0.0111(*)		4.5	1.0	5	0.0142 (*)
**EPSC 10%-90% risetime (ms)**									
$Cast^{+/+}/Elks^{+/+}$	0.62	0.04	6	control	t-test	0.72	0.08	6	control
$Cast^{-/-}/Elks^{-/-}$	0.61	0.05	5	0.8595(ns)		0.72	0.11	5	0.9714(ns)
**Ca^{2+} current amplitude (nA)**									
$Cast^{+/+}/Elks^{+/+}$	1.27	0.11	6	control	t-test	1.34	0.10	6	control
$Cast^{-/-}/Elks^{-/-}$	0.62	0.05	5	0.0006(***)		0.65	0.05	5	0.0003(***)
**Ca^{2+} charge (pC)**									
$Cast^{+/+}/Elks^{+/+}$	1.6	0.1	6	control	t-test	13.2	1.1	6	control
$Cast^{-/-}/Elks^{-/-}$	0.8	0.1	5	0.0007(***)		6.3	0.5	5	0.0004(***)
**EPSC charge/Ca^{2+} charge**									
$Cast^{+/+}/Elks^{+/+}$	14.2	2.0	6	control	t-test	3.93	0.7	6	control
$Cast^{-/-}/Elks^{-/-}$	11.7	2.4	5	0.4461 (ns)		3.2	0.6	5	0.4168 (ns)
**1 ms/10 ms EPSC ratio**									
$Cast^{+/+}/Elks^{+/+}$	1.02	0.04	6	control	Mann Whitney U test				
$Cast^{-/-}/Elks^{-/-}$	0.99	0.06	5	0.9307 (ns)					

***EPSC- excitatory postsynaptic current.**

Table S5. Related to Figure 5, mEPSC recordings and basal [Ca^2] in the presynaptic terminal.

Parameter (unit)	Mean [Median]	SEM	n	P	statistics
**Frequency (Hz)**					
Cast$^{+/+}$/*Elks*$^{+/+}$	1.49 [0.45]	0.65	12		Mann-Whitney test
Cast$^{-/-}$/*Elks*$^{-/-}$	1.8 [1.55]	0.28	14	0.0095 (**)	
**Amplitude (pA)**					
Cast$^{+/+}$/*Elks*$^{+/+}$	58.03 [56.75]	6.34	12		t-test
Cast$^{-/-}$/*Elks*$^{-/-}$	52.9 [56.48]	3.45	14	0.82 (ns)	
**Half-width (ms)**					
Cast$^{+/+}$/*Elks*$^{+/+}$	0.44 [0.43]	0.018	12	control	Mann-Whitney test
Cast$^{-/-}$/*Elks*$^{-/-}$	0.39 [0.4]	0.011	14	0.05(ns)	
**Frequency (Hz) in the presence of Cd^{2+}**					
before	1.25 [1.17]	0.2	10	control	Paired t-test
during	1.16 [1.01]	0.17	10	0.41 (ns)	
**basal Ca^{2+} concentration (nM)**					
Cast$^{+/+}$/*Elks*$^{+/+}$	85.1	5	7	control	t-test
Cast$^{+/+}$/*Elks*$^{-/-}$	74.3	6	9	0.2015 (ns)	
